# Unique Evolution of Antiviral Tetherin in Bats

**DOI:** 10.1128/jvi.01152-22

**Published:** 2022-09-29

**Authors:** Joshua A. Hayward, Mary Tachedjian, Adam Johnson, Aaron T. Irving, Tamsin B. Gordon, Jie Cui, Alexis Nicolas, Ina Smith, Victoria Boyd, Glenn A. Marsh, Michelle L. Baker, Lin-Fa Wang, Gilda Tachedjian

**Affiliations:** a Health Security Program, Life Sciences Discipline, Burnet Institute, Melbourne, Victoria, Australia; b Department of Microbiology, Monash University, Clayton, Victoria, Australia; c CSIRO, Australian Centre for Disease Preparedness, Health and Biosecurity Business Unit, Geelong, Victoria, Australia; d Zhejiang University-University of Edinburgh Institute, Zhejiang University School of Medicine, Zhejiang University International Campus, Haining, Zhejiang, China; e Second Affiliated Hospital, Zhejiang University School of Medicine, Hangzhou, Zhejiang, China; f Programme in Emerging Infectious Diseases, Duke-NUS Medical School, Singapore, Singapore; g CAS Key Laboratory of Molecular Virology & Immunology, Institut Pasteur of Shanghai, Chinese Academy of Sciences, Beijing, China; h CSIRO, Health and Biosecurity Business Unit, Acton, Australian Capital Territory, Australia; i Singhealth Duke-NUS Global Health Institute, Singapore, Singapore; j Department of Microbiology and Immunology, University of Melbourne, Peter Doherty Institute for Infection and Immunity, Melbourne, Victoria, Australia; Emory University School of Medicine

**Keywords:** antiviral, bats, BST-2, evolution, filoviruses, innate immunity, restriction factors, retroviruses, tetherin, viruses

## Abstract

Bats are recognized as important reservoirs of viruses deadly to other mammals, including humans. These infections are typically nonpathogenic in bats, raising questions about host response differences that might exist between bats and other mammals. Tetherin is a restriction factor which inhibits the release of a diverse range of viruses from host cells, including retroviruses, coronaviruses, filoviruses, and paramyxoviruses, some of which are deadly to humans and transmitted by bats. Here, we characterize the tetherin genes from 27 bat species, revealing that they have evolved under strong selective pressure, and that fruit bats and vesper bats express unique structural variants of the tetherin protein. Tetherin was widely and variably expressed across fruit bat tissue types and upregulated in spleen tissue when stimulated with Toll-like receptor agonists. The expression of two computationally predicted splice isoforms of fruit bat tetherin was verified. We identified an additional third unique splice isoform which includes a C-terminal region that is not homologous to known mammalian tetherin variants but was functionally capable of restricting the release of filoviral virus-like particles. We also report that vesper bats possess and express at least five tetherin genes, including structural variants, more than any other mammal reported to date. These findings support the hypothesis of differential antiviral gene evolution in bats relative to other mammals.

**IMPORTANCE** Bats are an important host of various viruses which are deadly to humans and other mammals but do not cause outward signs of illness in bats. Furthering our understanding of the unique features of the immune system of bats will shed light on how they tolerate viral infections, potentially informing novel antiviral strategies in humans and other animals. This study examines the antiviral protein tetherin, which prevents viral particles from escaping their host cell. Analysis of tetherin from 27 bat species reveals that it is under strong evolutionary pressure, and we show that multiple bat species have evolved to possess more tetherin genes than other mammals, some of which encode structurally unique tetherins capable of activity against different viral particles. These data suggest that bat tetherin plays a potentially broad and important role in the management of viral infections in bats.

## INTRODUCTION

Bats are reservoirs of viruses that are highly pathogenic to other mammals, including humans. Viruses such as Hendra, Nipah, Ebola, Marburg, severe acute respiratory syndrome coronaviruses (SARS-CoV-1 and likely also SARS-CoV-2), and the Sosuga virus have crossed species barriers from bats into humans (1–5). Laboratory studies have demonstrated that specific bat species can be infected with Ebola, Marburg, SARS-CoV-1, Hendra, and Nipah viruses without showing clinical signs of disease (6–10). Recently, this has led to efforts to elucidate whether there are differences between the antiviral strategies of bats and those of other mammals (11–19). These studies have identified that genes related to immunity in bats are under a significant degree of positive selection, in addition to differences in the copy number and diversity of innate immune genes of bats relative to other mammals (12–15, 17, 20).

Tetherin (BST-2, CD317) is a mammalian restriction factor that inhibits the release of diverse enveloped viral particles from the cells in which they are produced (21–23). These viruses include retroviruses, coronaviruses, filoviruses, and paramyxoviruses, some of which are transmissible by bats (24–27). Tetherin is a membrane-associated glycoprotein and its activity is determined by its structure rather than by its primary amino acid sequence (28, 29). In addition, tetherin possesses a secondary role in immune signaling events by triggering the NF-κB signaling pathway, leading to stimulation of the antiviral interferon response (30–33).

In humans, tetherin is expressed across most cell types, including its BST-2 namesake, bone marrow stromal cells, and its expression is upregulated by stimulation with type I interferons (34–36). Tetherin is a dimeric dual-anchor type II membrane protein that contains one protein anchor, a transmembrane domain near its N terminus, an extracellular coiled-coil domain, and a glycophosphatidylinositol (GPI) lipid anchor, which is attached to its C terminus as a post-translational modification (37–39). Tetherin contains a number of conserved cytosine and asparagine motifs within the extracellular domain, with respective roles in dimerization and glycosylation; and a dual-tyrosine motif (Y·X·Y) in its cytoplasmic region, which has roles in viral particle endocytosis and immune signaling cascades (31, 40). Tetherin is located in lipid rafts at the plasma membrane, where many viral particles bud during acquisition of their host membrane-derived viral envelopes (39). During the viral budding process, one anchor remains embedded in the nascent viral envelope while the other remains attached to the plasma membrane, tethering the virion to the cell and preventing its release into the extracellular environment (28, 38).

Tetherin is common to all mammals, and orthologs which share structural and functional similarity, but not sequence similarity, exist in other vertebrates (29). Most mammals carry only a single tetherin gene; however gene duplication has been observed in sheep, cattle, opossums, and wallabies (29, 41). Furthermore, human tetherin is expressed in two alternative isoforms (30), the long (l-tetherin) and short (s-tetherin) isoforms, which differ through the truncation of 12 amino acid (aa) residues at the N terminus of l-tetherin. Computational analysis of the genomes of megabats from the genus *Pteropus* predict two additional isoforms, X1 and X2, with an internal rather than an N-terminal difference in amino acid sequences, although whether these isoforms are expressed is unknown (12). The predicted isoform X1 is homologous to human l-tetherin, and the X2 isoform is a splice variant which contains a 7-aa exclusion within the extracellular coiled-coil domain relative to isoform X1. A previous analysis of tetherin in the fruit bats Hypsignathus monstrosus and Epomops buettikoferi revealed that these species expressed a homolog of *Pteropus* isoform X1 and that it is a functional restriction factor capable of inhibiting the release of the bat-hosted Nipah virus, as well as virus-like particles (VLPs) and GP-virosomes produced from the Ebola virus and the human immunodeficiency virus (23, 42).

We hypothesized that given the role of bats as hosts of pathogenic enveloped viruses, and the differences observed in other antiviral genes of their innate immune system (14), this diversity might also exist in their tetherin genes. Here, we report that fruit bats possess a unique structural isoform which has differential activity against VLPs, restricting filoviral but not retroviral VLPs, in addition to the two computational predictions, tetherin isoforms X1 and X2, which were generated through the automated NCBI annotation pipeline from the published Pteropus alecto genome (NCBI: PRJNA171993) (12). Adding to a previous report describing the presence and expression of three tetherin paralogs in the microbat Myotis daubentonii, and four tetherin genes in the genome of M. lucifugus (suborder Yangochiroptera) (18), we have found that microbats of the genus *Myotis* (e.g., *M. macropus*) possess at least five tetherin genes, two of which contain large structural differences in the extracellular coiled-coil domain. An analysis of the tetherin genes of 27 species of bats (including *P. alecto*) revealed that bat tetherin has been subjected to strong positive selection, indicating that these flying mammals have experienced evolutionary pressure on this antiviral gene. Collectively, our findings indicate that bats have undergone tetherin gene expansion and diversification relative to other mammals, supporting the hypothesis of differential antiviral gene evolution in bats.

## RESULTS

### Bats possess structural homologs of human tetherin.

**FIG 1:**
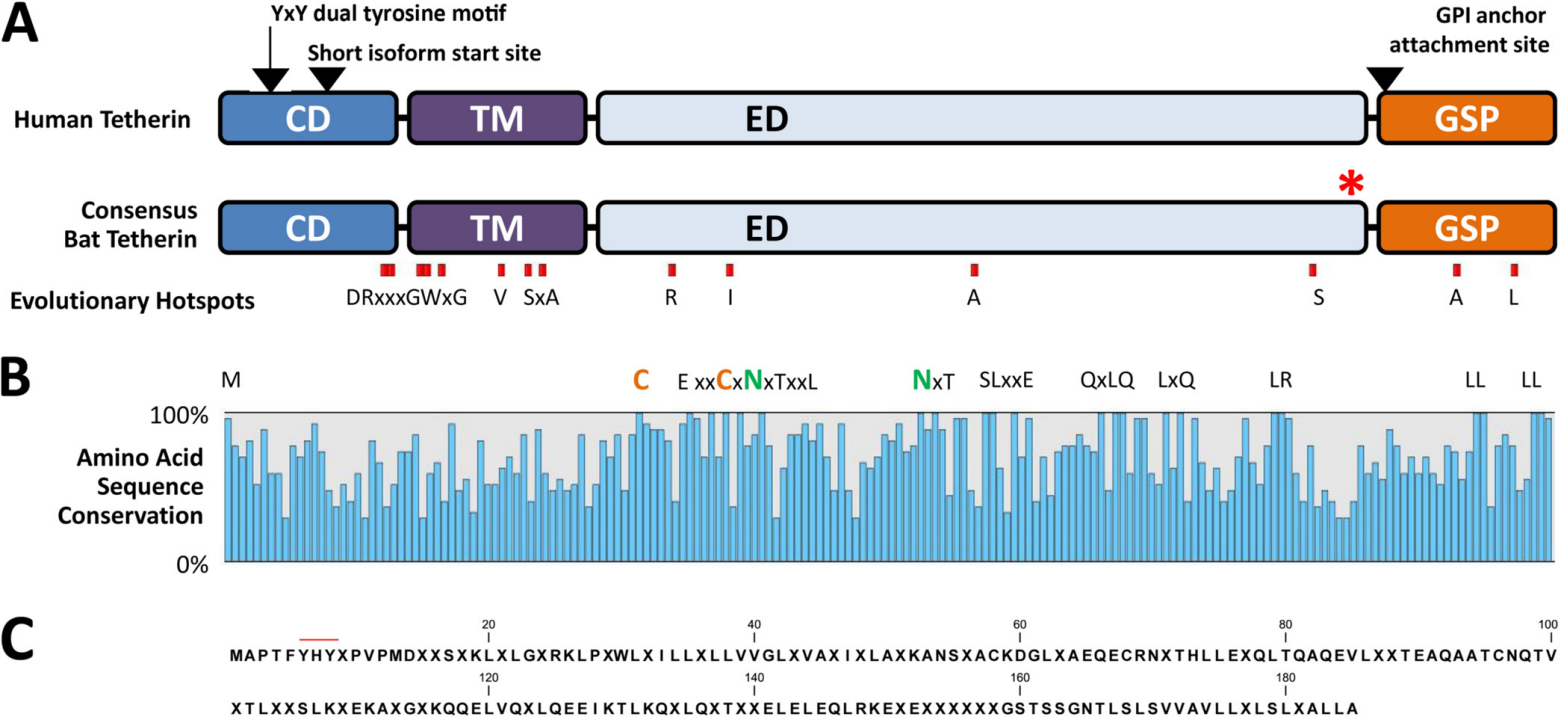
Bat tetherin structure and sequence diversity. (A) Consensus bat tetherin amino acid sequence structures and motifs generated through the multiple sequence alignment of tetherin from 27 bat species (Data Set S1) and compared to human tetherin. Amino acid residues under strong positive selection are denoted by red boxes. The Y·X·Y dual-tyrosine motif, the alternative start site for the short isoform of tetherin, and the GPI-anchor attachment site positions are indicated by arrows. Red asterisk (*) indicates location of the inserted molecular tags in the Pteropus alecto and Myotis macropus tetherin expression constructs. CD, cytoplasmic domain; TM, transmembrane domain; ED, extracellular domain; GPI, glycophosphatidylinositol; GSP, GPI signal peptide. (B) Sequence diversity among bat tetherin proteins is indicated by the percentage of amino acid sequence conservation at each site of the consensus bat tetherin. Amino acids conserved in all 27 sequences are represented by their letters. Amino acids represented by X are variable and are included to indicate the sequence distances between closely positioned conserved residues. (C) Consensus bat tetherin sequence. Amino acid residues represented in >50% of bat tetherin sequences are indicated with their letter. Positions at which no amino acid residue is represented in >50% of bat tetherin sequences are indicated with ‘X’. The red line indicates the position of the dual-tyrosine motif.

To advance our understanding of bat tetherin homologs, we mined all available publicly accessible sequence read archives representing 26 species of bats through a BLASTn analysis, using the *P. alecto* tetherin isoform A as the query sequence (Table 1). The species analyzed represent 8 of the 20 families within the order Chiroptera: three families (Hipposideridae, Pteropodidae, and Rhinolophidae) from the suborder Yinpterochiroptera, and five families (Emballonuridae, Miniopteridae, Molossidae, Vespertilionidae, and Phyllostomidae) from the suborder Yangochiroptera. This analysis revealed mRNA sequences representing homologs of human tetherin from 26 bat species, in addition to the homolog obtained from *P. alecto* cDNA, enabling the generation of consensus bat tetherin structural domains and amino acid sequence (Fig. 1A and C) from a multiple-sequence alignment (supplemental Data Set S1). The primary sequence lengths range from 177 to 221 aa, compared to 180 aa for human l-tetherin; of these, only 23 residues were conserved in 100% of the bat tetherin sequences analyzed (Fig. 1B). The assembled tetherin sequence for all bats was a homolog of human l-tetherin, except for the black-bearded tomb bat (Taphozous melanopogon), which was predicted to possess an equivalent of the human s-tetherin. The predicted *T. melanopogon* tetherin nucleotide sequence contains two key mutations upstream of its short isoform start site: an ATG to CTG change in the long isoform start codon and a TAG stop codon between the two start codon sites, either of which would be sufficient to prevent the production of the long isoform of tetherin. No nucleotide conflicts exist between the 17 reads mapped to this region of the *T. melanopogon* tetherin sequence, indicating that these mutations are unlikely to represent sequencing or consensus alignment errors.

**TABLE 1:** Sequence accessions with BioProject and cDNA sources for the prediction and/or confirmation of tetherin homologues

Source species	Common name	Sequence	GenBank accession no.	UniProt identifier	NCBI project accession no.	cDNA source
Bats						
Artibeus jamaicensis	Jamaican fruit-eating bat	Tetherin	MT274379		PRJNA61227	
Carollia brevicauda	Silky short-tailed bat	Tetherin	MT274380		PRJNA139591	
Carollia perspicillata	Seba’s short-tailed bat	Tetherin	MT274381		PRJNA291081	
Cynopterus sphinx	Indian short-nosed fruit bat	Tetherin	MT274382		PRJNA222415	
Desmodus rotundus	Common vampire bat	Tetherin	MT274383		PRJNA178123	
Eidolon helvum	Straw-colored fruit bat	Tetherin	MT274384		PRJNA209406	
Eonycteris spelaea	Cave nectar bat	Tetherin	MT274385		PRJNA255191	
Hipposideros armiger	Great roundleaf bat	Tetherin	MT274386		PRJNA260965	
Macrotus californicus	California leaf-nosed bat	Tetherin	MT274387		PRJNA226078	
Miniopterus natalensis	Natal long-fingered bat	Tetherin	MT274388		PRJNA270639	
Miniopterus schreibersii	Common bent-wing bat	Tetherin	MT274389		PRJNA218524	
		Tetherin	MT274405			Kidney cell line
Murina leucogaster	Hilgendorf’s tube-nosed bat	Tetherin	MT274390		PRJNA182766	
Myotis brandtii	Brandt’s bat	Tetherin	MT274391		PRJNA218631	
Myotis davidii	David’s myotis	Tetherin	MT274392		PRJNA172130	
Myotis laniger	Chinese water myotis	Tetherin	MT274393		PRJNA255191	
Myotis lucifugus	Little brown bat	Tetherin	MT274394		PRJNA246229	
Myotis macropus	Large-footed myotis bat	Tetherin A	MT274406			Kidney cell line
		Tetherin B	MT274407			Kidney cell line
		Tetherin C Isoform A	MT274408			Kidney cell line
		Tetherin C isoform B	MT274409			Kidney cell line
		Tetherin D	MT274410			Kidney cell line
		Tetherin E	MT274411			Kidney cell line
Myotis myotis	Greater mouse-eared bat	Tetherin	MT274395		PRJNA267654	
Myotis ricketti	Rickett’s big-footed bat	Tetherin A	MT274396		PRJNA198831	
		Tetherin A	MT274412			Spleen tissue
		Tetherin B	MT274413			Spleen tissue
Pteropus alecto	Black flying fox	Tetherin isoform A	MT274397		PRJNA73831	
		Tetherin isoform A	MT274414			Spleen tissue
		Tetherin isoform B	MT274415			Spleen tissue
		Tetherin isoform C	MT274416			Spleen tissue
Pteropus vampyrus	Large flying fox	Tetherin	MT274398		PRJNA20325	
Rhinolophus ferrumequinum	Greater horseshoe bat	Tetherin	MT274399		PRJNA231230	
Rhinolophus macrotis	Big-eared horseshoe bat	Tetherin	MT274400		PRJNA261657	
Rousettus aegyptiacus	Egyptian fruit bat	Tetherin	MT274401		PRJNA300284	
Tadarida brasiliensis	Mexican free-tailed bat	Tetherin	MT274402		PRJNA184055	
Taphozous melanopogon	Black-bearded tomb bat	Tetherin	MT274403		PRJNA255191	
Uroderma bilobatum	Tent-making bat	Tetherin	MT274404		PRJNA268573	
Other mammals						
Bos taurus	Cow	Tetherin A isoform A		J7M5G6		
		Tetherin A isoform B		J7MAQ7		
		Tetherin B		J7M2B2		
Felis catus	Cat	Tetherin		F8R0X8		
Homo sapiens	Human	L-tetherin		Q10589-1		
		S-tetherin		Q10589-2		
Loxodonta africana	Elephant	Tetherin		G3UKK9		
Macaca mulatta	Rhesus macaque	Tetherin		C4P4A2		
Mus musculus	Mouse	Tetherin		Q8R2Q8		
Ovis aries	Sheep	Tetherin A		D5JZS7		
		Tetherin B		D5JZS8		
Pan troglodytes	Chimpanzee	Tetherin		D7RVC2		

The dual-tyrosine Y·X·Y motif, which is critical for mediating viral-particle endocytosis and is involved in immune signaling (31, 40), is variable among bat species and exists in different combinations of Y|C·X·Y|H (Fig. 1A and C). All bat species possess at least one tyrosine residue within this motif (Fig. 1C). Conservation of the protein domain organization and key structural motifs of bat tetherin, despite significant amino acid sequence diversity, supports the present understanding of tetherin as a protein whose functions are mediated through structural rather than sequence-mediated interactions (28).

### Bat tetherin genes are under significant positive selection.

**FIG 2:**
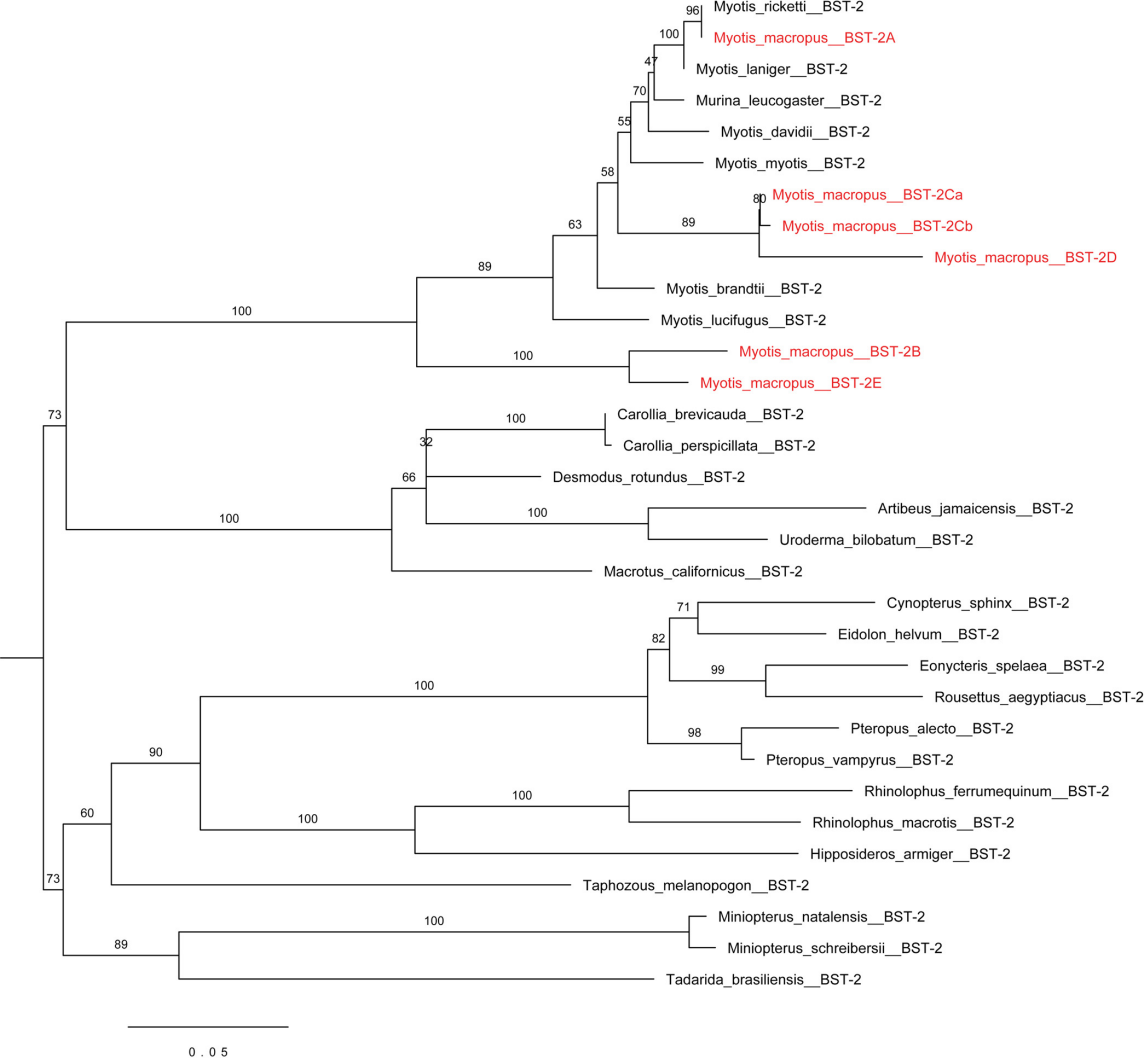
Maximum likelihood phylogenetic analysis of bat tetherin (BST-2) nucleotide sequences. Phylogeny was generated with CODEML implemented in PAML4 software (69). Input tree was generated based on phylogeny generated by TimeTree (70). Nodes are labeled with the name of the bat species from which the tetherin sequences are derived. Multiple tetherin paralogs are represented for *Myotis macropus*, labeled in red. Scale bar represents the number of nucleotide substitutions per site.

To assess the evolutionary pressure applied during the speciation of bats, a selection test was performed to analyze bat tetherin genes spanning to ~65 million years ago (mya) in chiropteran history. First, to avoid the inclusion of paralogs in the selection test, we performed a phylogenetic analysis of the tetherin sequences (Fig. 2). Based on branch lengths and branching patterns, Myotis macropus tetherin 2A (BST-2A) was determined to be the ortholog of tetherin in other bat species. *M. macropus* tetherins B to E were considered to represent paralogs and excluded from the subsequent selection test. The selection test revealed that overall, bat tetherin genes have been subjected to positive selection, with an average ratio of non-synonymous (dN) to synonymous (dS) mutations, dN/dS, of 1.125 over the chiropteran phylogeny, with numerous specific positions subjected to a large degree of positive selection, with dN/dS values of 2.497 to 2.561 (Table 2). These sites are predominantly located in the transmembrane domain and the regions of the cytoplasmic and extracellular domains immediately adjacent to it (Fig. 1A). This analysis suggests the presence of evolutionary pressure exerted on tetherin, by viral antagonists or countermeasures that target residues within tetherin, following the speciation of the order Chiroptera. The apparent ubiquity of tetherin among bats, in concert with significant sequence diversity and evidence of strong positive selection pressures operating on each species, supports the notion that tetherin plays a major role in the antiviral repertoire of bats.

**TABLE 2:** Evolutionary hot spots in bat tetherin

Tetherin amino acid^*a*^	Pr(w > 1)^*b*^	dN/dS^*c*^
D24	0.987^*d*^	2.538
R25	0.972^*d*^	2.513
G29	0.994^*e*^	2.551
W30	0.972^*d*^	2.513
G32	1.000^*e*^	2.561
V39	0.973^*d*^	2.515
S43	1.000^*e*^	2.561
A45	0.994^*e*^	2.551
R63	1.000^*e*^	2.56
I71	0.990^*d*^	2.543
A105	0.962^*d*^	2.497
S151	0.973^*d*^	2.514
A171	0.999^*e*^	2.558
L179	0.988^*d*^	2.541

a The consensus amino acid identity is indicated at the position number relative to the Cynopterus sphinx tetherin protein sequence.

b Positively selected sites.

c Ratio of non-synonymous (dN) to synonymous (dS) mutations.

d *P* > 95%.

e *P* > 99%.

### Fruit bats possess unique structural isoforms of the tetherin protein generated through alternative splicing of a single tetherin gene.

**FIG 3:**
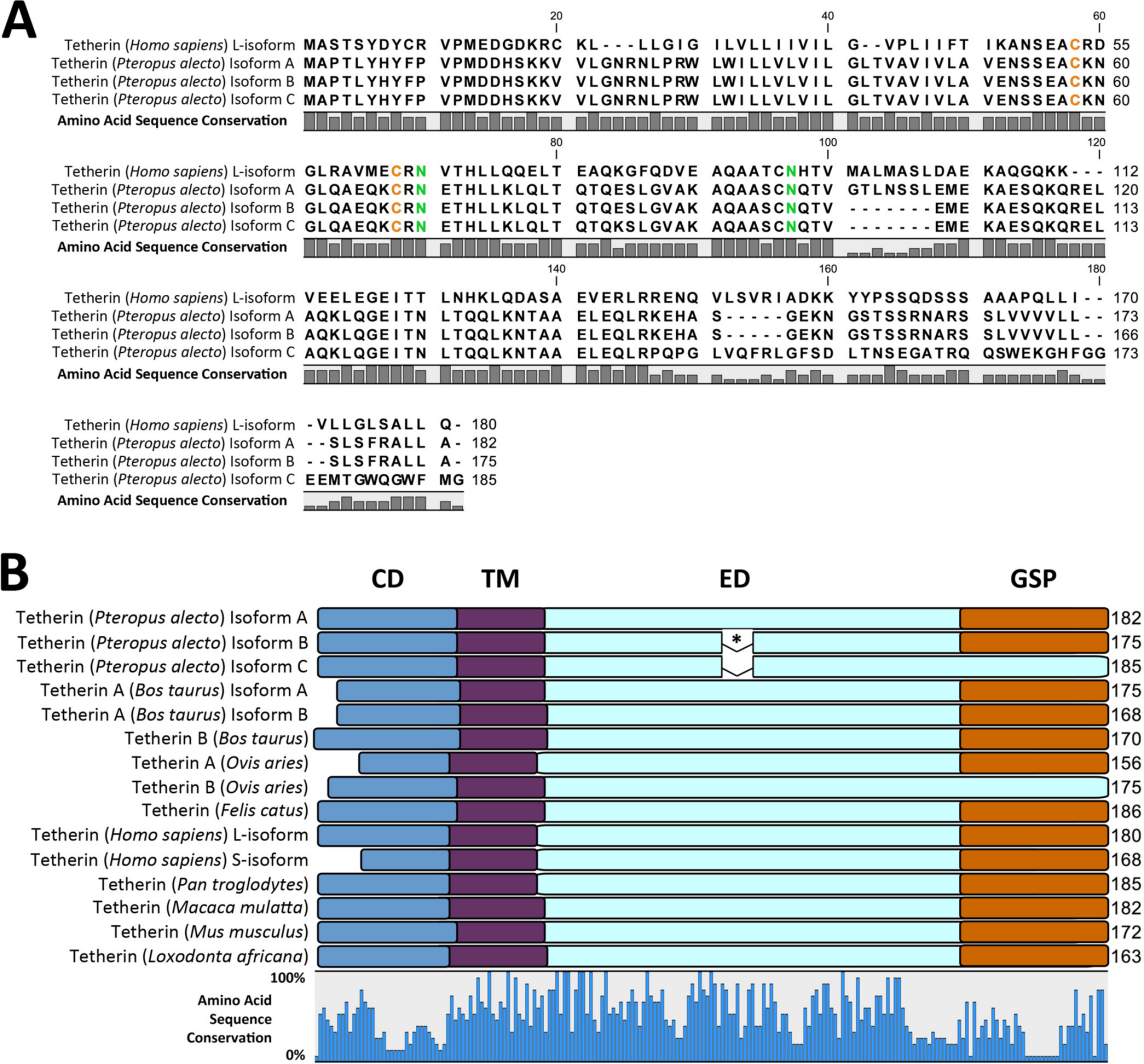
Multiple sequence alignment (MSA) and protein domains of mammalian tetherin amino acid sequences. (A) MSA reveals amino acid residues differing between human and *P. alecto* sequences, indicated by the amino acid sequence conservation bar graph below the sequences. Conserved cysteine and asparagine residues are colored orange and green, respectively. (B) Protein domains of mammalian tetherin proteins are depicted as overlays of an MSA. Sequence conservation is indicated by the bar graph below the alignment. Protein domains are color coded: CD, cytoplasmic domain (blue); TM, transmembrane domain (purple); ED, extracellular domain (light blue); GSP, glycophosphatidylinositol signal peptide (orange). Asterisk (*) indicates a 7-amino acid (aa) exclusion in *P. alecto* tetherin isoforms B and C relative to isoform A.

The expression of the fruit bat tetherin gene was initially assessed by a BLASTn search within the transcriptome of an Australian fruit bat, the black flying fox (Pteropus alecto) using the human tetherin amino acid sequence as the search query. We identified 30 contigs matching the query (lowest E value = 9.21 × 10^−23^). A single *P. alecto* homolog of tetherin, here described as isoform A, homologous to human l-tetherin and the predicted X1 isoform (GenBank ID: XM_006904279), was identified. *P. alecto* tetherin isoform A has low primary amino acid sequence conservation (37%) compared to human l-tetherin (Fig. 3A) and other mammalian (28% to 47%) tetherin sequences which are homologous to human l-tetherin (Fig. 3B). Despite the low amino acid sequence identity, the predicted secondary structures and protein domains are largely conserved between bats and other mammals (Fig. 3B). All previously reported tetherin proteins contain the cytoplasmic (CD), transmembrane (TM), and extracellular domains (ED), and the post-translationally cleaved GPI-anchor signal peptide (GSP); with the exception of sheep tetherin B, which does not contain the GSP domain.

To verify the expression of bat tetherin isoform A, a cDNA library was prepared from *P. alecto* spleen tissue, comprising several immune cell types, and assessed for the presence of transcripts matching that identified in the *P. alecto* transcriptome. Primers were designed to flank the open reading frame of tetherin isoform A, and amplicons were generated by PCR. Amplicons were then cloned into plasmid vectors for DNA sequencing. This analysis revealed two additional isoforms of tetherin, isoforms B and C. Isoform B was homologous to the computationally predicted isoform X2 (GenBank ID: XM_015587122). Both isoforms B and C were predicted to contain structural differences relative to isoform A. They both possess a 7-aa exclusion in the middle of the extracellular domain, while isoform C was additionally predicted to contain an alternative C terminus without the GSP domain (Fig. 3B). The absence of a GSP domain suggests that it is unlikely that parallel homodimers of isoform C possess the capacity to tether viral particles to the cell.

**FIG 4:**
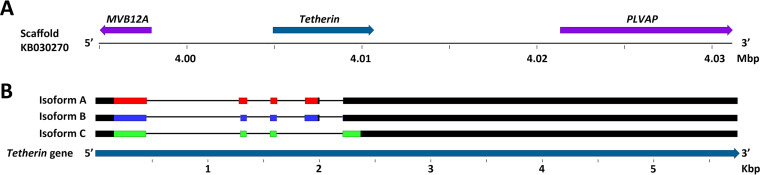
Mapping the tetherin gene to the *P. alecto* genome. (A) Tetherin gene location (dark blue) and neighboring genes (purple). Arrows indicate the orientation of each gene’s protein coding domain sequence. Sequence numbers and gene orientations are relative to the beginning of the gene scaffold in the 5′ to 3′ direction. (B) Tetherin mRNA exons are shown and color-coded: isoform A, red; isoform B, blue; isoform C, green. Exons are mapped to scale against the tetherin gene. Sequence numbers are relative to the beginning of the gene. The 5′ and 3′ untranslated regions of the mRNA are colored black.

Mapping the tetherin cDNA sequences against the *P. alecto* genome identified a single tetherin gene located on scaffold KB030270 (Fig. 4A), between the MVB12A (multivesicular body subunit 12A) and PLVAP (plasmalemma vesicle-associated protein) genes, similar to the human tetherin gene (viewable in the Ensembl genome browser: http://www.ensembl.org/Homo_sapiens/Location/View?db=core;g=ENSG00000130303;r=19:17402886-17405683). *P. alecto* isoform B and C mRNA were generated using an alternative splice acceptor site for the first intron, resulting in the 7-aa exclusion, while the distinct C terminus of isoform C is the result of alternative mRNA splicing which incorporates exon 5 while excluding exon 4, which was used in isoform A and B (Fig. 4B).

### Vesper bats possess multiple tetherin genes that contain unique structural variations.

To further investigate the diversity of tetherin in non-fruit bat species, we amplified the tetherin nucleotide sequences for the vesper bat species Myotis ricketti and Miniopterus schreibersii using primers which were designed on the basis of the transcriptome sequences (Table 3). These primers were used for PCR of cDNA samples generated from *M. ricketti* spleen tissue and a *M. schreibersii* kidney cell line to amplify the tetherin coding domain sequences (Table 3). Primers for *M. ricketti* were also used to amplify the tetherin-coding region from cDNA generated from a Myotis macropus kidney cell line as it was reasoned that since these species belong to the same genus, primers designed for one might be capable of amplifying tetherin from the other. Following PCR, amplified DNA from *M. ricketti*, *M. macropus*, and *M. schreibersii* were gel-purified, cloned, and sequenced.

**TABLE 3:** cDNA amplification and vector sequencing primers for fruit bat and vesper bat tetherins^*a*^

Tetherin source and type	cDNA amplification primer	Primer sequence (5′ > 3′)	Direction	Target region	Successful combinations	PCR T_A_ (°C)
*Pteropus alecto*						
Tetherin	PaF1_JH_119	TCACTGCAAGGGGTTCTCTC	Forward	5′ UTR	119/121−2	60
	PaF2_JH_120	GGAAACTTCACTGCAAGGGG	Forward	5′ UTR	120/121−2	60
	PaR1_JH_121	CTTCTCCCAGCTTTGTTGCC	Reverse	3′ UTR		
	PaR2_JH_122	CTCCTCTCCCCCAAAATGTC	Reverse	3′ UTR		
*Miniopterus schreibersii*						
Tetherin	MiniF1_JH_466	CCCACAAACTCCCTACACCC	Forward	5′ UTR	466/468	60
	MiniF2_JH_467	ATGCTAATGAAGGGGCGGGG	Forward	5′ UTR	466/469	62
	MiniR1_JH_468	CTGTCTGTCTTCCTGGGAC	Reverse	3′ UTR	467/469	62
	MiniR2_JH_469	GGACAGGTCAGGGAAACCAA	Reverse	3′ UTR		
*Myotis macropus* and *Myotis ricketti*						
Tetherins A, C, and D	MyoF1_JH_479	TCCACTGCATCCCTCTG	Forward	5′ UTR	479/481−4	60
	MyoF2_JH_480	ATGGCACCCACTTTTTAC	Forward	5′ UTR	480/481−4	60
	MyoR1_JH_481	TCAGCCAGGTTAGAATGTG	Reverse	3′ UTR		
	MyoR2_JH_482	TCCTTGGGCAAACAGCTCTC	Reverse	3′ UTR		
	MyoR3_JH_483	CAGGAAACTCTCAGAAAAG	Reverse	3′ UTR		
	MyoR4_JH_484	CATCTTTCCAAGACCACA	Reverse	3′ UTR		
Tetherins B and E	MyoF3_JH_474	GCTCCTGTGCATCCCTCTGG	Forward	5′ UTR	474/478	60
	MyoR5_JH_478	CCTGGTTAGAATGTGCTTT	Reverse	3′ UTR		

a Primers used to amplify tetherin homologues from cDNA generated from *Myotis macropus*, *M. ricketti*, and Miniopterus schreibersii. Combinations of forward and reverse primers identified as capable of amplifying tetherin are indicated alongside the highest annealing temperature at which amplification was successful. UTR, untranslated region; *T_A_*, annealing temperature.

**FIG 5:**
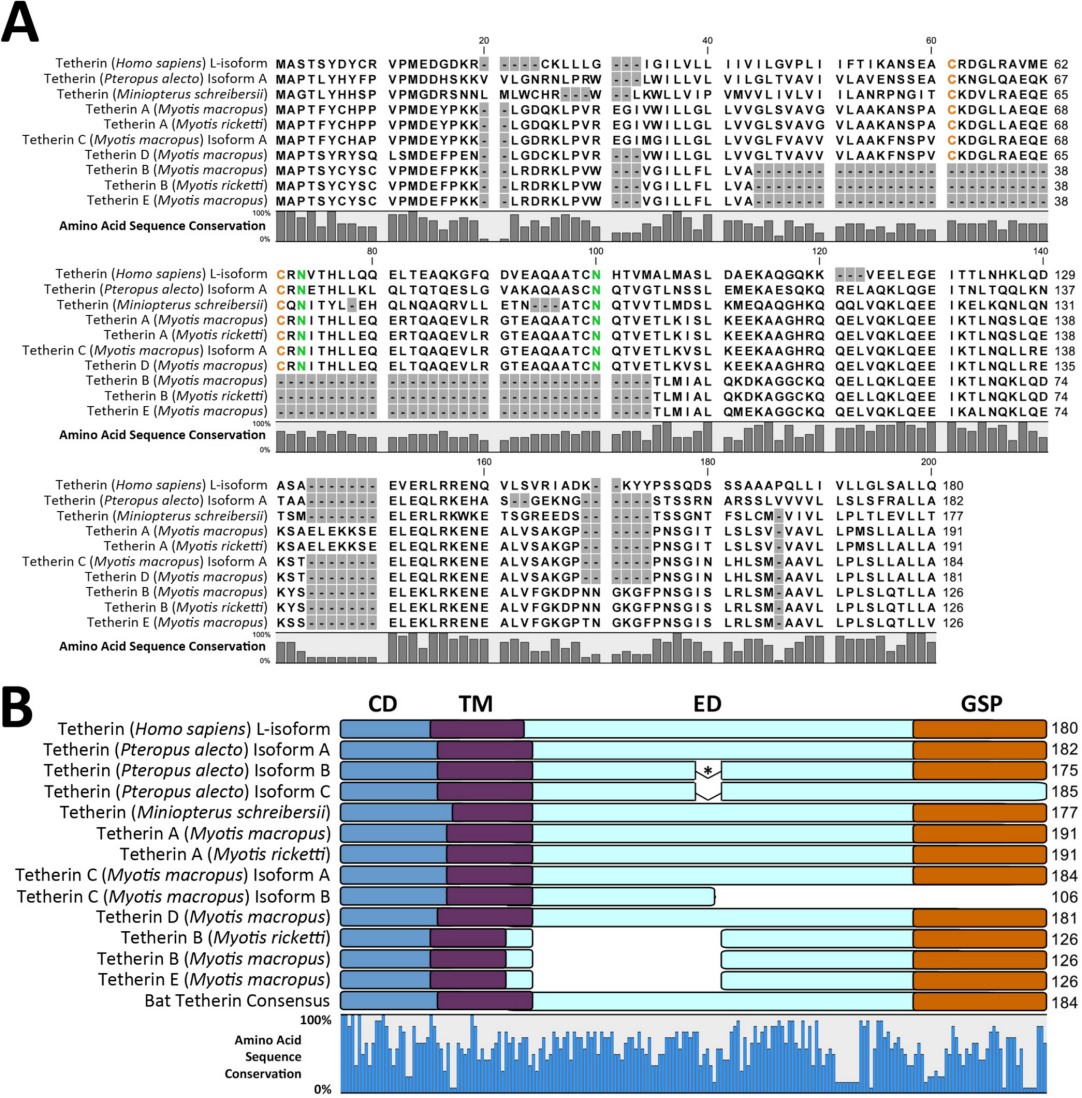
MSA and protein domains of fruit bat and vesper bat tetherin amino acid sequences. (A) Percentage of sequence conservation is indicated by a bar plot below the MSA. Gap positions are shaded in gray. Conserved cysteine and asparagine residues are colored orange and green, respectively. (B) Protein domains of bat tetherin proteins are depicted as overlays of an MSA. Amino acid sequence conservation is indicated by the bar graph below the alignment. Protein domains are indicated and color coded as follows: CD, cytoplasmic domain (blue); TM, transmembrane domain (purple); ED, extracellular domain (light blue); GSP, glycophosphatidylinositol signal peptide (orange). An asterisk (*) indicates the 7-aa exclusion in *P. alecto* tetherin isoforms B and C relative to isoform A.

These microbats were found to express various tetherin homologs which included unique structural variations (Fig. 5A and B). The tetherin homolog of human l-tetherin predicted for *M. schreibersii* was detected (Fig. 5A). Five unique tetherin variants differing in their encoded amino acid sequences (tetherin A to E) were identified for *M. macropus*, two of which, tetherin A and B, were also detected in *M. ricketti* (Fig. 5A). These tetherin variants encode three distinct homologs (tetherin A, C, and D) of human l-tetherin, sharing the same protein domains but differing in their amino acid sequence (Fig. 5).

An additional splice variant (isoform B) of *M. macropus* tetherin C was also identified. *M. macropus* tetherin C isoform B is predicted to have a C-terminal truncation and possesses only the cytoplasmic domain, transmembrane domain, and less than half of the extracellular domain (Fig. 5B). Accordingly, it is predicted to lack the GPI signal peptide required for the post-translational addition of a GPI-anchor, as was also found for *P. alecto* tetherin isoform C (Fig. 3B). The absence of a GPI anchor indicates that it is unlikely that homodimers of *M. macropus* tetherin C isoform B possess the capacity to tether viral particles to the cell. *M. macropus* tetherins B and E were found to be structurally unique, possessing a large deletion of ~60 amino acids in the extracellular coiled-coil domain (Fig. 5B) compared to the other tetherin homologs, which has been shown to be critical for viral particle restriction (28, 43). This deletion results in the exclusion of the conserved disulfide bond-forming cysteine residues and asparagine-linked glycosylation sites, indicating that this form of tetherin is unlikely to form dimers or be glycosylated in the manner of human tetherin (28). They are, however, predicted to possess all other tetherin domains, including the transmembrane domain and GPI signal peptide, indicating that they may still be capable of tethering viral particles. *M. macropus* tetherins B and E share 92.1% amino acid sequence identity, indicating they are the products of a relatively recent gene duplication.

**FIG 6:**
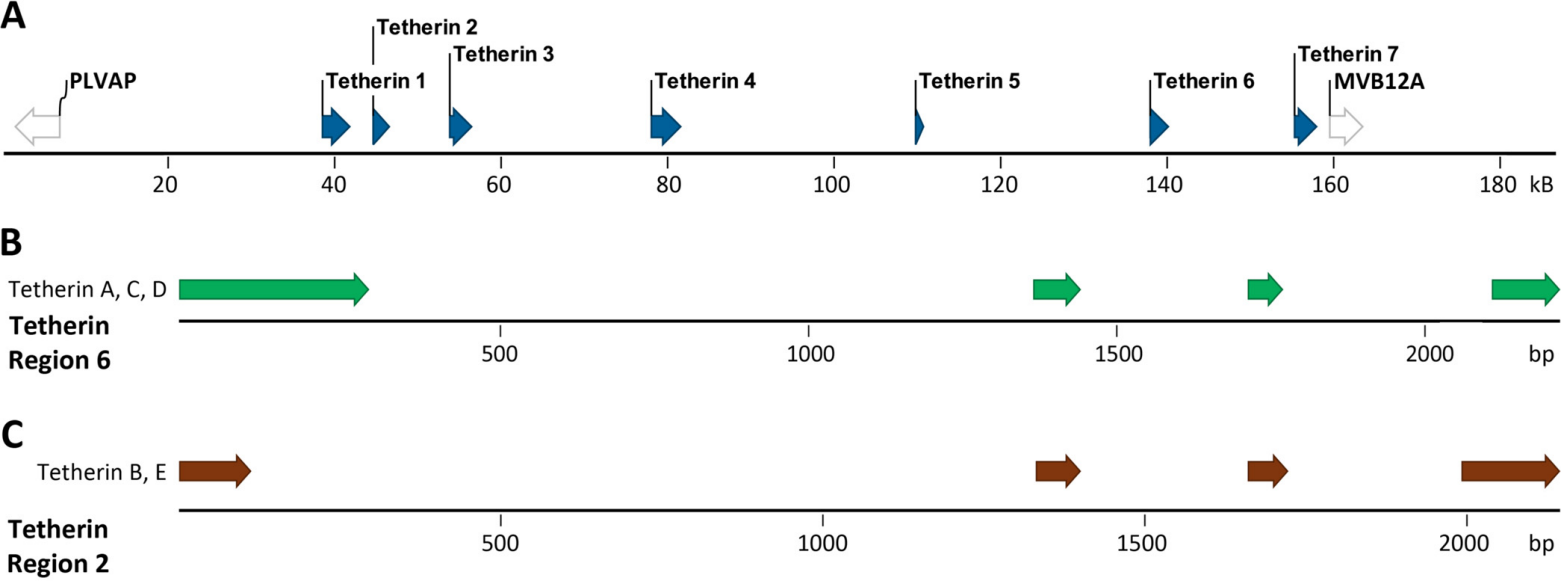
Mapping the tetherin genes of *M. macropus* to the *M. lucifugus* genome. Tetherin sequences derived from *M. macropus* cDNA mapped against the publicly available genome of M. lucifugus (Myoluc v2.0 genome assembly). (A) The tetherin gene locus of M. lucifugus is located within scaffold GL430608, flanked by the PLVAP and MVB12A genes. (B) *M. macropus* tetherins A, C, and D mapped against M. lucifugus tetherin region 6. (C) *M. macropus* tetherins B and E mapped against M. lucifugus tetherin region 2.

While there is no available genome for *M. macropus*, the most closely related publicly accessible genome assemblies are for the relatively distantly related Myotis lucifugus and M. davidii, which diverged from *M. macropus* approximately 19 and 14 mya, respectively (44, 45). For comparison, humans and chimpanzees diverged approximately 6.5 mya (46). A BLASTn analysis of the *M. macropus* tetherin nucleotide sequences against the M. davidii and M. lucifugus genomes revealed first that both species were too distantly related to *M. macropus* to match each *M. macropus* gene product specific to the M. davidii or M. lucifugus gene regions. However, the M. lucifugus genome assembly included a single gene scaffold (GL430608) which contained matches to all query *M. macropus* tetherin sequences, comprising seven potential M. lucifugus tetherin genes (Fig. 6A) and indicating the presence of a tetherin gene locus within this scaffold. Further analysis of the mapped exons, as described below, enabled confirmation of the M. lucifugus tetherin regions. The M. lucifugus tetherin gene locus was located between the MVB12A and PLVAP genes, as observed for the single *P. alecto* tetherin gene (Fig. 4 and 6).

The genome assembly of M. davidii also contained significant matches to *M. macropus* tetherin sequences; however, these were spread across seven different gene scaffolds against which no *M. macropus* tetherin coding domain sequence could be matched in its entirety. For this reason, further genomic analysis of *Myotis* tetherin genes was confined to the M. lucifugus genome.

The M. lucifugus tetherin gene locus (Fig. 6A) contains seven potential genes that are defined by the presence of two or more exons mapping to the *M. macropus* tetherin cDNA. Three exons across tetherin gene regions 1, 2, and 6 possess all of the coding exons required to express homologs to *M. macropus* tetherins A, C, and D (Fig. 6B). Furthermore, M. lucifugus tetherin gene regions 2 and 3 possess all of the coding exons required to produce homologs to *M. macropus* tetherins B and E (Fig. 6C). These data indicate that microbats of the genus *Myotis* possess a greater number and diversity of tetherin genes than any mammal reported to date.

These findings expand upon a recent report by Hölzer et al. (18) which identified the expression of three tetherin paralogs, upregulated by interferon treatment, in the vesper bat, *M. daubentonii*. Mapping of these paralogs against the genome of M. lucifugus revealed four tetherin genes, currently listed as novel gene predictions within the Ensembl database: ENSMLUG00000023691, ENSMLUG00000029243, ENSMLUG00000026989, and ENSMLUG00000023562 (18). Here, these genes are labeled tetherins 1, 2, 6, and 7, respectively (Fig. 6A).

### Tetherin expression pattern in *P. alecto* tissue samples.

**FIG 7:**
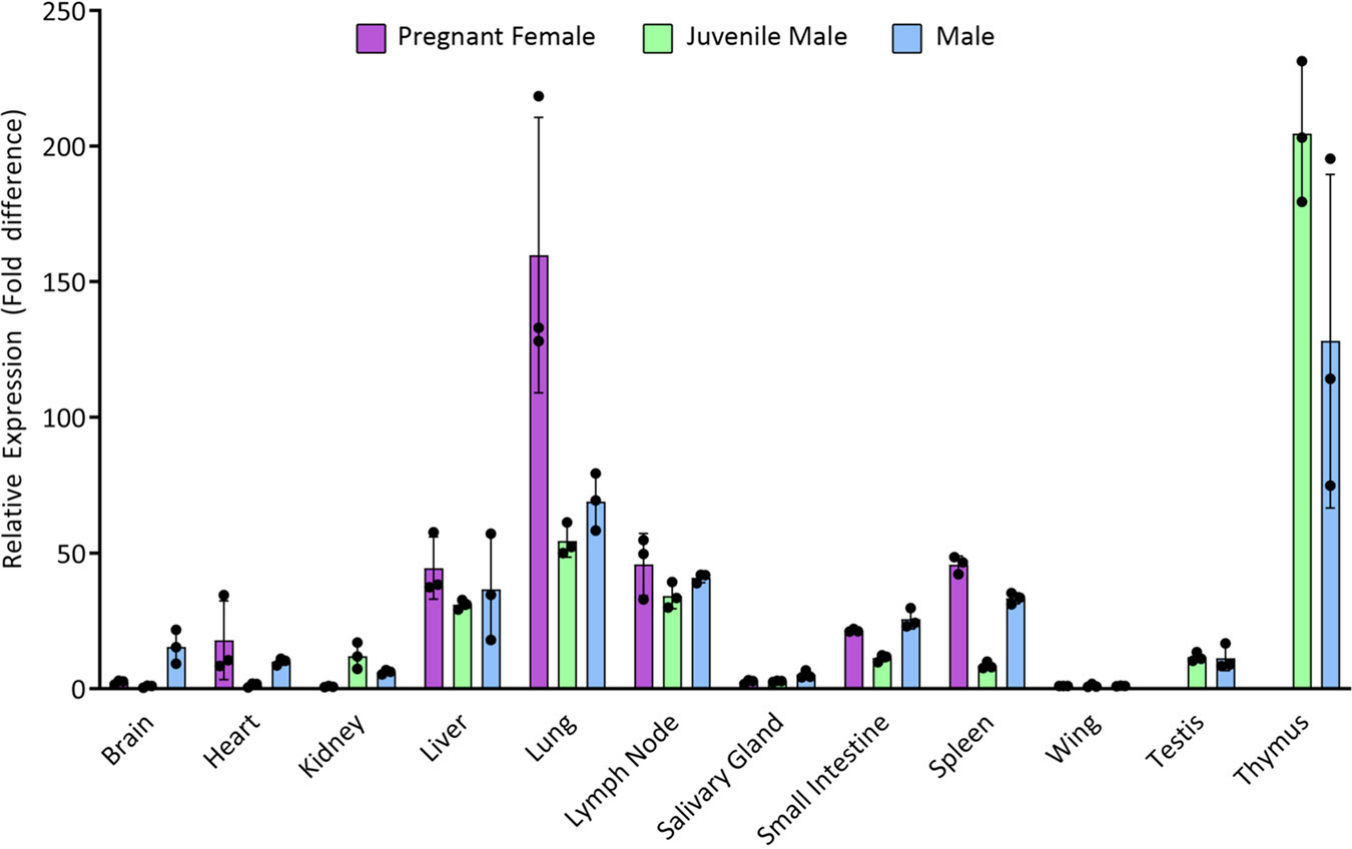
Relative tetherin expression across tissues from *P. alecto*. Expression of tetherin mRNA assessed by quantitative real-time PCR (qRT-PCR) analysis of tissues from three bats. In all cases, cycle threshold (*C_T_*) values for tetherin were normalized against expression of the 18S rRNA gene. For all individuals, relative tetherin expression in each tissue is calibrated against expression in wing tissue. A thymus sample was not obtained for the female bat. Error bars represent the standard error (*n* = 3).

Tetherin is widely and variably expressed across human tissues and is upregulated in response to type I interferon (35, 36). To assess the relative expression of bat tetherin across different tissue types, tissue samples were obtained from three individual *P. alecto* bats: a male, a pregnant female, and a juvenile male. Using primers which amplify all three isoforms of *P. alecto* tetherin, expression was analyzed by quantitative PCR (qPCR) with *C_T_* values normalized against the 18S rRNA housekeeping gene (Fig. 7). The two male bats exhibited similar tetherin expression patterns, with greatest levels in the thymus. A thymus tissue sample was not obtained from the female, although a similar pattern to the male bats was observed among most other tissues. The female bat had higher expression in the lung compared to the male bats, possibly reflecting an active response to a respiratory infection, an artifact of hormonal regulation, or natural heterogeneity between bats.

**FIG 8:**
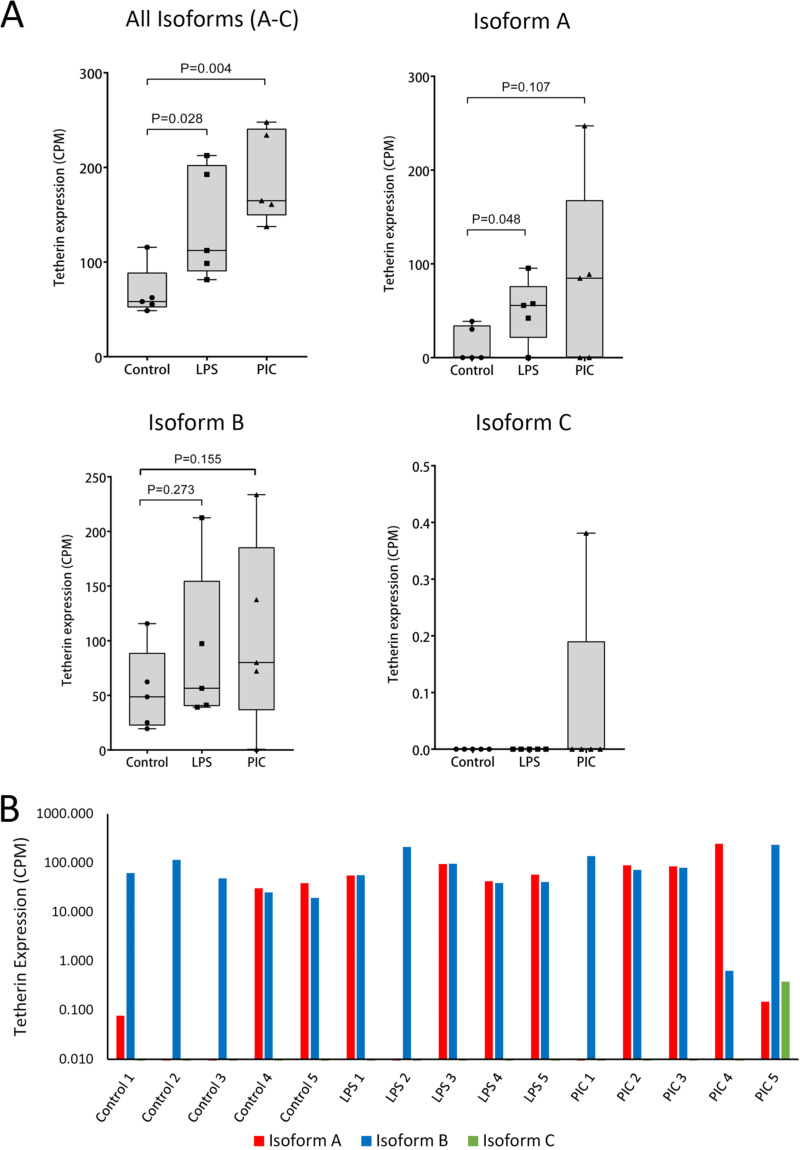
Expression of alternative isoforms of tetherin in stimulated and unstimulated *P. alecto* spleen tissue. Spleen tissue from 15 individuals were unstimulated, or stimulated with lipopolysaccharide (LPS) or polyinosinic:poly(C) (PIC) (*n* = 5 for each treatment). Tetherin expression was determined from RNA-seq sequence reads mapped against the *P. alecto* genomic scaffold, KB030270, which harbors the tetherin gene. Tetherin expression levels are represented as normalized counts-per-million (CPM) reads. (A) Tetherin expression by isoform. *P* values were determined using a one-tailed Mann-Whitney U test. (B) Expression of tetherin isoform in each individual sample.

Previous studies in vesper bats have demonstrated that tetherin is upregulated in response to type I interferon alpha stimulation (18). In *P. alecto*, lipopolysaccharide (LPS) treatment increases the percentage of B cells in the spleen (47), and polyinosinic:poly(C) (PIC) treatment is observed to increase the expression of the interferon-stimulated gene *ISG54* (48). To determine if pathogen-mediated stimulation can upregulate tetherin expression in fruit bats, we analyzed the transcriptome of *P. alecto* spleen tissue from animals treated *in vivo* with the Toll-like receptor (TLR) agonists LPS or PIC. We found that *P. alecto* tetherin was significantly upregulated by LPS (*P* = 0.028) and PIC (*P* = 0.004) (Fig. 8A).

We next assessed if there was a difference in the expression of the alternative tetherin isoforms A, B, and C, and the impact on the expression of each isoform following treatment with the TLR agonists LPS and PIC. This analysis revealed that tetherin is predominantly expressed as isoform B (14/15 samples; Fig. 8B), while stimulation with LPS significantly increased the expression of isoform A (*P* = 0.048), but not that of isoforms B or C (Fig. 8A). Stimulation with PIC increased the expression of isoforms A and B compared to unstimulated tissue; however, this effect was variable among individual bat spleen samples and did not reach significance. Isoform C expression was only observed in a single PIC-treated spleen sample. These data show that tetherin isoforms are expressed in tissue and can be upregulated by stimulation with TLR-agonists.

### *P. alecto* tetherin protein expression and cellular localization.

To characterize bat tetherin, we analyzed tetherin from two Australian bats, the fruit bat *P. alecto* and the vesper bat *M. macropus*. The coding sequences of the *P. alecto* tetherin isoforms A, B, and C and *M. macropus* tetherins A and B were cloned into mammalian expression plasmids. While numerous vesper bat tetherins were identified in this study, *M. macropus* tetherins A and B were selected for further analysis because tetherin A is a homolog of human l-tetherin and tetherin B was the most structurally unique, possessing a large deletion within the extracellular coiled-coil domain compared to tetherin A (Fig. 5B).

All tetherin coding sequences were modified with the addition of a hemagglutinin (HA) tag sequence (N-SG**YPYDVPDYA**GS-C) to enable antibody-based detection of protein expression. In all cases, the HA tag was inserted at the position immediately following the end of the coiled-coil region of the extracellular domain (Fig. 1A), which is the equivalent location to that previously utilized in the tagging of human tetherin (28).

**FIG 9:**
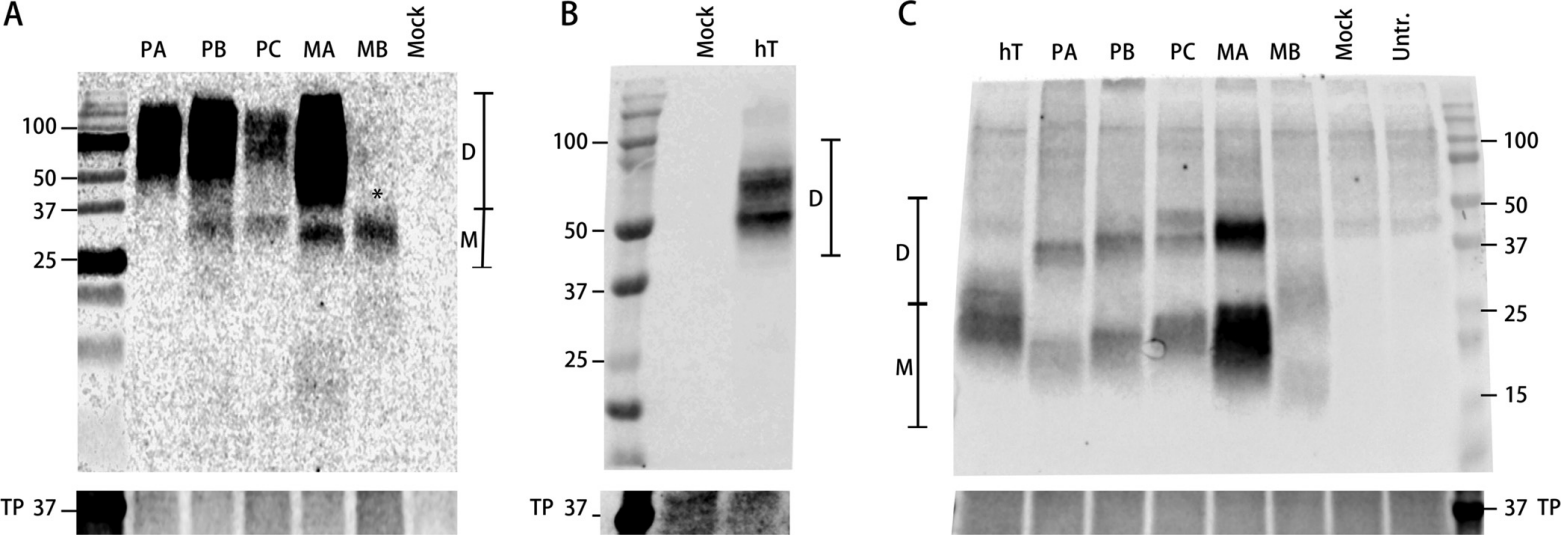
SDS-PAGE and Western blot analysis of the expression of modified bat tetherin constructs. Western blot analysis of the expression of hemagglutinin (HA)-tagged Pteropus alecto tetherin isoforms A, B, and C (PA, PB, and PC, respectively), *M. macropus* tetherins A and B (MA and MB, respectively), and human tetherin (hT) compared against cells mock-transfected (Mock) with an equivalent mass of empty vector plasmid pcDNA3.1 and untransfected (Untr) cells. Tetherin was expressed in mammalian HEK293T cells, and cell lysates were collected 48 h after transfection with expression plasmids. (A) Bat tetherin expression. SDS-PAGE of cell lysates run on a 12% polyacrylamide gel under non-reducing conditions. (B) Human tetherin expression. SDS-PAGE of cell lysates run on an Any kD gradient polyacrylamide gel (Bio-Rad) under non-reducing conditions. (C) SDS-PAGE of cell lysates treated with PNGase to deglycosylate proteins, reduced in the presence of dithiothreitol, and run on an Any kD polyacrylamide gel (Bio-Rad). Tetherin is present in dimeric (D) and monomeric (M) forms. *The band present in MB represents a dimer rather than a monomer due to the relatively smaller predicted size of monomeric *M. macropus* tetherin B (~14.5 kDa). Protein size is expressed in kDa. Total protein (TP) stain was used as a loading control.

To evaluate tetherin protein expression, mammalian HEK293T cells, which do not express tetherin in the absence of type I interferon stimulation (21), were transfected with bat tetherin expression constructs. Cell lysates were extracted using a method for enhanced extraction of GPI-anchored proteins (49) and tetherin expression was observed by non-reducing SDS-PAGE and Western blot analysis (Fig. 9A and B). The predicted molecular weights of unglycosylated and GPI-anchored tetherin proteins are listed in Table 4. Human tetherin is glycosylated, and the *P. alecto* and *M. macropus* tetherins analyzed here are predicted to contain several glycosylation sites by the GlycoMine prediction tool (50) (Table 5). For all isoforms of *P. alecto* tetherin and *M. macropus* tetherin A, the predicted glycosylation sites with the highest probability are the highly conserved N-linked glycosylation sites highlighted in Fig. 3A and Fig. 5A. *M. macropus* tetherin B contains a 60-aa exclusion that spans both conserved glycosylation sites (Fig. 5A); however, it contains two additional predicted glycosylation sites, one of which is an O-linked glycan, with a probability score of >0.5, indicating that it is likely to be glycosylated at these sites. All predicted glycosylation sites with a prediction probability score of >0.5 are listed in Table 5.

**TABLE 4:** Predicted molecular mass for unglycosylated tetherin monomers and dimers

Unglycosylated tetherin	Predicted molecular mass (kDa)					
	Human tetherin	*P. alecto*			*M. macropus*	
		Tetherin iso A	Tetherin iso B	Tetherin iso C^*a*^	Tetherin A	Tetherin B
Monomeric, HA-tagged, GSP-cleaved + GPI anchor	20.8	20.1	19.4	22.1	21.3	14.5
Dimeric, HA-tagged, GSP-cleaved + GPI anchor	41.6	40.2	38.8	44.2	42.6	29

a Molecular mass calculations for *P. alecto* tetherin isoform C do not involve GSP-cleavage or GPI-anchor addition. HA, hemagglutinin; GPI, glycophosphoinositol; GSP, GPI signal peptide.

**TABLE 5:** Predicted glycosylation sites with a probability score of >0.5, calculated by GlycoMine

Tetherin	Rank	Position	Site	Motif	Score	Type
Human						
L-tetherin	1	49	N	IIFTIKANSEACRDG	1	N-linked
	1	124	N	EGEITTLNHKLQDAS	1	N-linked
	1	141	N	VERLRRENQVLSVRI	1	N-linked
	1	65	N	RAVMECRNVTHLLQQ	1	N-linked
	1	92	N	EAQAATCNHTVMALM	1	N-linked
*Pteropus alecto*						
Isoform A	1	97	N	KAQAASCNQTVGTLK	0.558	N-linked
	2	70	N	QAEQKCRNKTHLLEL	0.551	N-linked
	1	129	T	QKLQGEITNLTQQLK	0.507	O-linked
Isoform B	1	70	N	QAEQKCRNETHLLKL	0.538	N-linked
	2	97	N	KAQAASCNQTVEMEK	0.52	N-linked
	1	122	T	QKLQGEITNLTQQLK	0.507	O-linked
Isoform C	1	70	N	QAEQKCRNETHLLKL	0.538	N-linked
	2	97	N	KAQAASCNQTVEMEK	0.52	N-linked
	1	122	T	QKLQGEITNLTQQLK	0.507	O-linked
*Myotis macropus*						
Tetherin A	1	71	N	LAEQECRNITHLLEQ	0.718	N-linked
	2	98	N	EAQAATCNQTVETLK	0.675	N-linked
	3	55	N	GVLAAKANSPACKDG	0.558	N-linked
	4	133	N	QEEIKTLNQSLQEKS	0.535	N-linked
	1	140	S	NQSLQEKSAELEKKS	0.563	O-linked
Tetherin B	1	69	N	QEEIKTLNQKLQDKY	0.526	N-linked
	1	77	S	QKLQDKYSELEKLRK	0.544	O-linked

**FIG 10:**
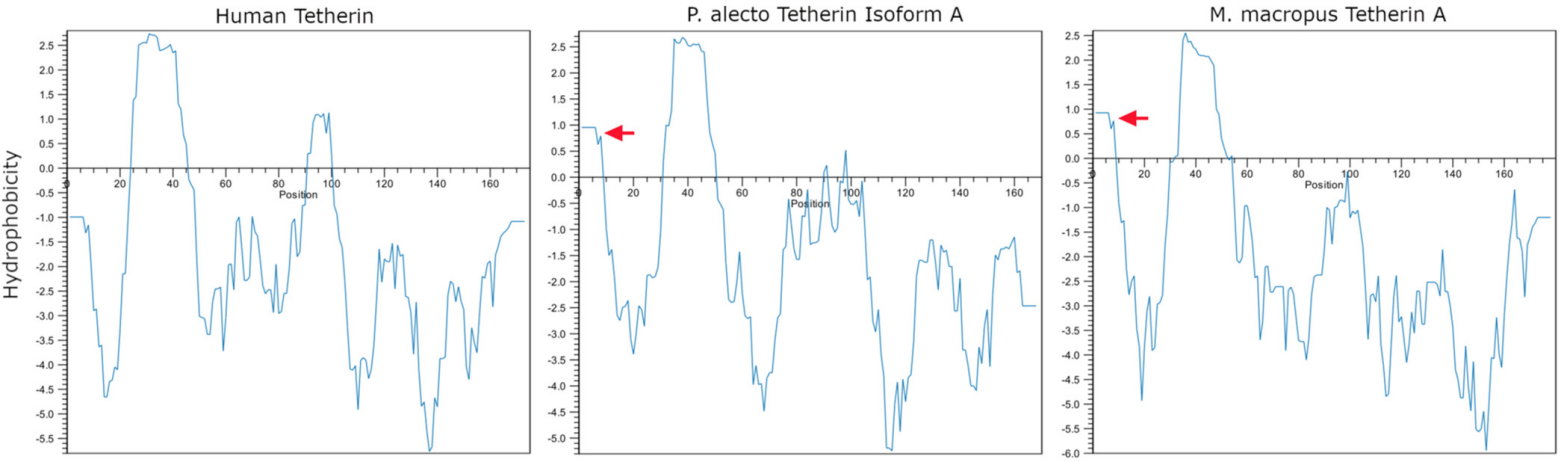
Hydrophobicity plot of human and bat tetherins. Amino acid sequences representing HA-tagged, GSP-cleaved human tetherin, *Pteropus alecto* tetherin isoform A, and *M. macropus* tetherin A were analyzed using the CLC hydrophobicity tool. Hydrophobicity was analyzed across the sequence with an 11-aa sliding window. Positive hydrophobicity scores indicate hydrophobic regions. Red arrows indicate the small region of hydrophobicity at the N termini of bat tetherins.

*P. alecto* tetherin isoforms A and B were detected primarily as broad bands at the expected positions for dimeric tetherin of ~56 to 75 kDa (Fig. 9A), which is consistent with human tetherin (Fig. 9B). *P. alecto* tetherin isoform C was present as both dimers and monomers (~32 to 35 kDa), and some monomers were also detected for isoform B (Fig. 9A). *M. macropus* tetherin A was also primarily detected as dimers, with some monomers also present (Fig. 9A). In contrast, *M. macropus* tetherin B, which is a smaller ~14.5-kDa protein relative to tetherin A (Table 4), was detected predominately as a dimer (~30 kDa) (Fig. 9A). The presence of broad/multiple bands in close proximity to each other likely reflects variable levels of tetherin glycosylation, as has been previously reported for human tetherin (28). To better resolve bat tetherin, we subjected samples to deglycosyation and a reducing agent (Fig. 9C). Consistent with the expected effect, deglycosylated tetherins migrated at lower molecular weights: ~39 to 48 kDa for dimers and ~15 to 24 kDa for monomers. Reduced and deglycosylated *M. macropus* tetherin B migrated as two bands, with the higher (~30 kDa) corresponding to partially glycosylated dimers and the lower (~15 kDa) to partially glycosylated monomers of tetherin (Table 4). The partial deglycosylation of this tetherin is possibly due to the presence of a predicted O-linked glycan which is not susceptible to PNGase F treatment (Fig. 9C; Table 5). Interestingly, bat tetherin was resistant to reducing conditions, in contrast to human tetherin (Fig. 9C), with only a partial decrease in bat tetherin dimers observed under conditions that almost fully reduced human tetherin to monomers. Because protein dimers may be stabilized by hydrophobic interactions, we analyzed the hydrophobicity of the tetherin proteins *in silico* using the hydrophobicity tool in CLC Genomics Workbench (Fig. 10). These data indicated that both *P. alecto* tetherin and *M. macropu*s tetherin A were predicted to contain a more hydrophobic N terminus (aa position 1 to 10) than human tetherin. This may account for the enhanced dimer stability observed here (Fig. 9C). Together, these data show that HA-tagged tetherin can be expressed and that these proteins differ in their ability to dimerize.

**FIG 11:**
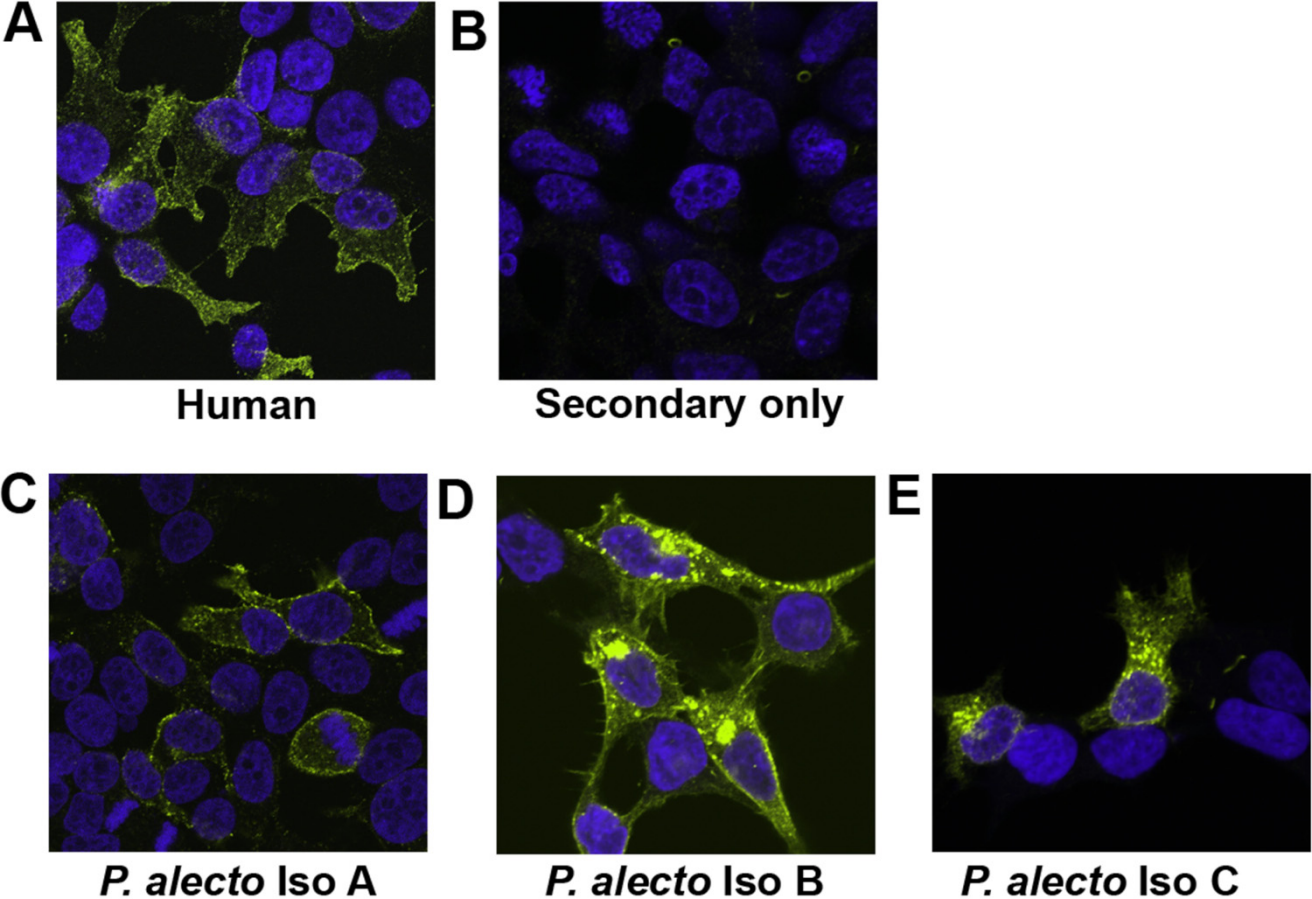
Expression of HA-tagged tetherin proteins. Human and *Pteropus alecto* tetherin isoforms A, B, and C in HEK293T cells. Tetherin localization in cells was detected using anti-HA-tag rabbit monoclonal Ig and anti-rabbit Alexa Fluor 488 secondary Ig. Nuclei are blue-stained with Hoechst. Fixed and permeabilized cells were imaged on a Nikon AR1 confocal microscope. (A) Human l-tetherin; (B) negative control, HEK293T cells treated without the inclusion of the primary antibody; (C) *P. alecto* tetherin isoform A; (D) *P. alecto* tetherin isoform B; (E) *P. alecto* tetherin isoform C. HA, hemagglutinin.

Human tetherin localizes to the plasma membrane at sites of virus budding, in addition to membranes of the trans-Golgi network and recycling compartments (21, 51). To determine localization of *P. alecto* tetherin isoforms, HEK293T cells were transfected with constructs expressing HA-tagged tetherin that was visualized by immunofluorescence. *P. alecto* tetherin isoform A localized to the plasma membrane, displaying a similar cellular localization pattern as human tetherin (Fig. 11A and C), while isoform C was predominantly localized in the cytoplasm (Fig. 11E). *P. alecto* tetherin isoform B demonstrated a fluorescence pattern with features shared by both isoforms A and C (Fig. 11D). These data show that bat tetherin isoforms can be expressed with isoforms A and B, but not C predominately localizing at the plasma membrane.

### *P. alecto* and *M. macropus* tetherin proteins display distinct ability to restrict HIVΔVpu virus-like particles.

**FIG 12:**
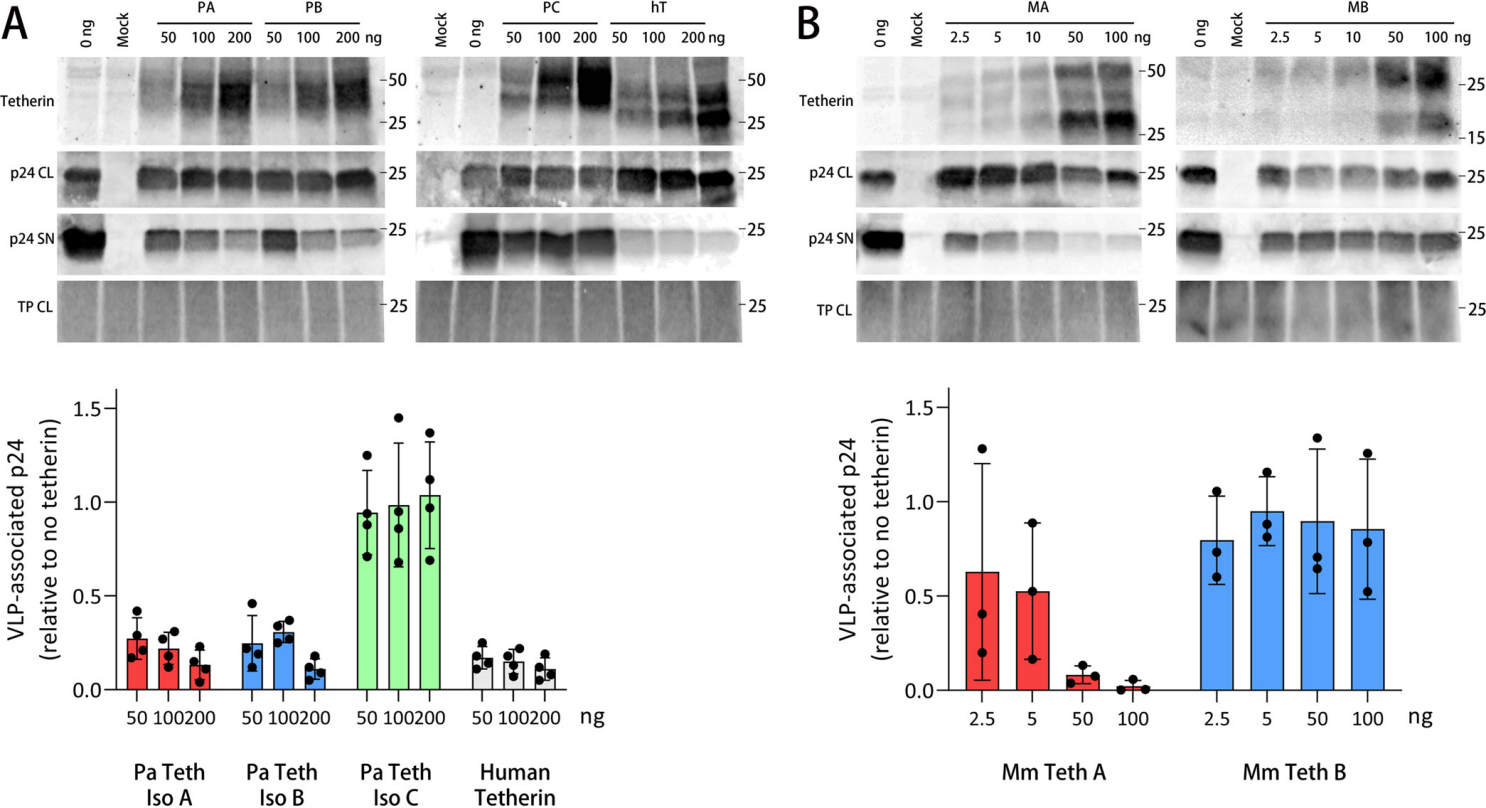
Bat tetherin restricts the release of HIV-1 virus-like particles (VLPs). (A) *P. alecto* tetherin isoforms A (PA), B (PB), C (PC), or human tetherin, co-transfected with a HIVΔVpu construct. (B) *M. macropus* tetherin A (MA) and tetherin B (MB) co-transfected with a HIVΔVpu construct. Mammalian HEK293T cells were co-transfected with 200 ng of the HIVΔVpu plasmid expression construct encoding the HIV Gag-Pol polyprotein, which generates HIV-1 VLPs that do not include the tetherin antagonist Vpu, and 0 to 200 ng of the tetherin plasmid expression vector. VLPs were harvested at 48 h and concentrated by ultracentrifugation using a sucrose cushion. VLP and cell lysates (not deglycosylated) were subjected to reducing SDS-PAGE and Western blot analysis. HIV-1 VLPs were detected with a mouse anti-p24 primary antibody and goat anti-mouse Alexa Fluor 680 fluorophore-conjugated fluorescent secondary antibody. Tetherin was detected with a rabbit anti-HA primary antibody and goat anti-rabbit Alexa-Fluor 800 fluorophore-conjugated fluorescent secondary antibody. Representative Western blots are shown. Protein molecular weight is expressed in kDa. Total protein stain was used as a loading control. The extent of VLP restriction was quantitated by densitometric analysis of Western blots comparing the relative ratios of VLPs present in the viral lysates and cell lysates from *n* = 4 (*P. alecto*) or *n* = 3 (*M. macropus*) independent assays. Error bars represent the standard deviation. HIV-1, human immunodeficiency virus type 1; SN, cell culture supernatant; CL, cell culture lysate; TP, total protein stain.

Tetherin from humans and other mammals, including cats, sheep, and other bat species (Hypsignathus monstrosus and Epomops buettikoferi) restrict the release of HIV-1 VLPs from cells (23, 52, 53). To examine if tetherins from *P. alecto* and *M. macropus* function to similarly block viral particle release, we assessed their ability to restrict HIV-1 VLPs. HEK293T cells were co-transfected with constructs expressing bat tetherin and HIVΔVpu VLPs, which do not express Vpu, an antagonist of human tetherin (21). *P. alecto* tetherin isoforms A and B inhibited the release of HIVΔVpu VLPs, in contrast to tetherin isoform C which failed to block VLP release (Fig. 12A). *M. macropus* tetherin A also restricted HIVΔVpu VLP release from HEK293T cells; however, tetherin B was unable to inhibit HIVΔVpu VLP egress (Fig. 12B). These data indicate that, except for *P. alecto* tetherin isoform C and *M. macropus* tetherin B, HA-tagged bat tetherin proteins are functionally capable of restricting the release of HIV-1 particles from mammalian cells in the absence of Vpu.

### The structurally unique *P. alecto* tetherin isoform C restricts the release of filoviral virus-like particles.

**FIG 13:**
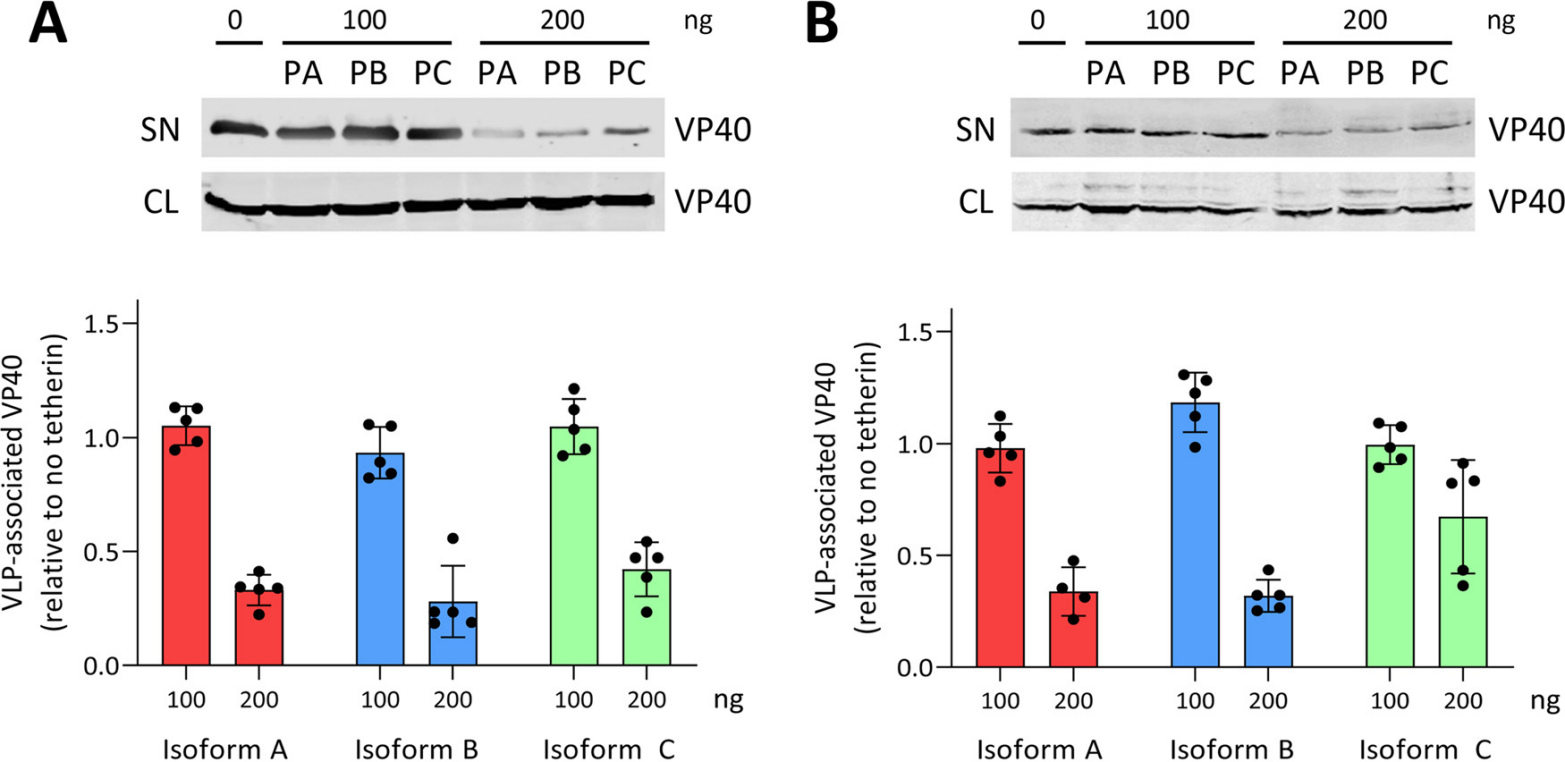
*Pteropus alecto* tetherin restricts the release of Ebola and Marburg virus-like particles (VLPs). *P. alecto* tetherin isoforms A (PA), B (PB), and C (PC), co-transfected with an (A) Ebola or (B) Marburg virus construct. Mammalian HEK293T cells were co-transfected with 200 ng of Ebola or Marburg virus plasmid expression construct encoding a VP40-eGFP protein which generates VLPs, and 0 to 200 ng of the tetherin plasmid expression vector. VLPs were harvested at 48 h and concentrated by ultracentrifugation through a sucrose gradient. VLP and cell lysates were subjected to SDS-PAGE and Western blot analysis. VLPs were detected with a mouse anti-GFP 4B10 primary antibody and a goat anti-mouse Alexa Fluor 680 fluorophore-conjugated fluorescent secondary antibody. Representative Western blots are shown. The extent of VLP restriction was quantitated by densitometric analysis of Western blots comparing the relative ratios of VLPs present in the viral lysates and cell lysates from *n* = 5 independent assays. Error bars represent the standard deviation. A nonparametric Wilcoxon rank-sum test was performed to calculate the statistical significance of the restriction of Ebola and Marburg VLPs in the 200 ng tetherin isoform A, B, and C treatment groups; *P* < 0.01 in all cases. SN, cell culture supernatant; CL, cell culture lysate.

We next investigated whether *P. alecto* tetherin isomers can restrict filovirus VLPs, including isoform C, which contains a unique C-terminal domain compared to isoforms A and B (Fig. 3B). In contrast to experiments with HIV-1 VLP, tetherin isoform C was able to restrict the release of VLPs composed of Ebola and Marburg virus VP40 matrix proteins (*P* = 0.008 for both; Fig. 13A and B). Ebola VLPs were restricted by isoforms A, B, and C to similar extents (Fig. 13A), while Marburg VLPs were restricted by isoform C to a lesser extent than by isoforms A and B (Fig. 13B). These data demonstrate that all *P. alecto* isomers were able to restrict filoviral VLPs, including isomer C which lacks the GSP domain.

## DISCUSSION

Bats are increasingly being recognized as hosts of viruses with zoonotic potential, driving efforts to better understand these host-virus relationships and the evolutionary features of bats that differentiate them from other mammals (14, 17, 26, 54). It has been hypothesized that the evolutionary adaptation to flight, including changes to the DNA-damage response, increased metabolic rates, and higher body temperatures have influenced the immune system of bats in such a way as to make them ideal hosts for viruses (12, 55, 56). To determine the differences between the innate antiviral defenses of bats relative to other mammals, we analyzed the genes and expressed transcripts of tetherin from diverse genera and species within the order Chiroptera. We found that in all but one species, bats possess genes that express transcripts encoding a tetherin protein homologous to the long isoform of human tetherin (l-tetherin). In addition, we found that *P. alecto* expresses three isoforms from a single tetherin gene, and that vesper bats (genus *Myotis*) encode five, possibly as many as seven, distinct tetherin genes.

Our findings expand on those of Hölzer et al. (18). The three tetherin paralog transcripts identified in *M. daubentonii* correspond to the four paralog genes identified in M. lucifugus. The findings of Hölzer et al. match our own, as the four genes identified by their group were the tetherin gene regions 1, 2, 6, and 7, described in this study. Regions 2 and 6 represent the two different structures observed among *M. macropus* tetherin (Fig. 5). Our study expands on these findings in that we describe the expression of five paralogs in *M. macropus* and the identification of seven potential tetherin genes within the tetherin gene locus of M. lucifugus. Additionally, we have identified important structural differences between the *Myotis* tetherin paralogs.

The only bat species which lacked the human l-tetherin homolog, *T. melanopogon*, possessed an equivalent of the short isoform of human tetherin (s-tetherin), an observation also reported for cats (57). In a study of feline tetherin, short isoform-exclusivity was found to improve viral restriction and decrease sensitivity to tetherin antagonism by HIV-1 Vpu compared to an engineered long isoform version of feline tetherin (57). Conversely, the feline immunodeficiency virus (FIV), which is adapted to the feline host, is not restricted by either long or short isoforms of feline tetherin (57). In humans, l-tetherin acts as a virus sensor which induces NF-κB signaling, in contrast to s-tetherin, which lacks the dual-tyrosine motif required to elicit this innate immune response (30, 31). The findings presented here suggest that if bat tetherin proteins are confirmed to similarly promote cytoplasmic domain-mediated immune signaling, then *T. melanopogon*, which only encodes a homolog of s-tetherin, would lack this function.

Bat tetherin amino acid sequences were found to be highly variable. The predicted protein lengths range from 177 to 221 aa (human l-tetherin is 180 aa) and of these, only 23 amino acid residues were conserved in 100% of the 27 bat tetherin sequences analyzed (Fig. 1B). Among these are the structurally important cysteine and asparagine residues which are responsible for tetherin dimerization and glycosylation, respectively (58, 59). The dual-tyrosine motif, responsible for mediating viral particle endocytosis and immune signaling (31, 40), was found to exist as a variable Y|C·X·Y|H motif across the bat tetherin variants analyzed. All bats maintained at least one of the two tyrosine residues. This observation is significant because mutational studies of human tetherin have demonstrated that the dual tyrosines provide redundancy for both the endocytic and signaling activities, which are maintained as long as either tyrosine is present (31, 40).

To understand the evolutionary selective pressures on tetherin genes across bat species, a selection test was performed which revealed that bat tetherin genes are under strong positive selection, with amino acid positions in and around the transmembrane domain being the region under the strongest selective pressure (Table 2 and Fig. 1A). These data are similar to previous reports that primate tetherins possess multiple sites under positive selection in this same region (28). The driver of positive selection in the case of primate tetherin genes is antagonism by viral countermeasures. These include HIV-1 Vpu (60) and SIV Nef (61), although it is likely that the anti-HIV-1 role of Vpu evolved too recently to have influenced the evolution of human tetherin (61). Because virus-host pairs and their respective restriction factors and countermeasures can differ between species within a mammalian clade, as is the case among primates (60, 61), sites under positive selection in one species may not be under the same pressure in other species. Reflecting this, one analysis of primate tetherin found that it was subject to neutral evolution overall, with episodic adaptive evolution within discrete primate lineages (62). Until more is understood regarding the specific interactions between the tetherin genes of various bat species and the viruses that infect them, we cannot be certain of the drivers of positive selection for bat tetherin. However, it is reasonable to hypothesize that, as for primates, viral antagonists of bat tetherin are likely to exert a large influence.

Venkatesh et al. (63) demonstrated that the configuration which tetherin dimers adopt during viral particle retention primarily consists of the GPI-anchor being embedded in the viral envelope and the transmembrane domain remaining attached to the cellular membrane. If this paradigm holds true for bat tetherin, then it follows that the tetherin-cell membrane interface is the major site of tetherin antagonism in bats, and it would be reasonable to speculate that the drivers of this selection are viral antagonists analogous in their mode of interaction, if not their structure or function, to lentiviral tetherin antagonists.

We amplified tetherin from spleen-derived cDNA of an Australian fruit bat, *P. alecto*, and confirmed the expression of the two computationally predicted splice variants (isoforms A [X1] and B [X2]), and additionally identified the expression of a third tetherin isoform, isoform C. Mapped against the *P. alecto* genome, all three isoforms were derived from the alternative splicing of a single tetherin gene (Fig. 3B). *P. alecto* tetherin isoform B possesses a 7-aa exclusion within the extracellular coiled-coil domain relative to isoform A, while isoform C is predicted to harbor the same 7-aa exclusion and an alternative C terminus lacking the GSP domain predicted to be present in tetherin isoforms A and B, which is necessary for the post-translational addition of the GPI-anchor. This is important because studies of human tetherin have demonstrated that the presence of a GPI-anchor is essential for restricting the release of viral particles (28, 43). The protein encoded by the gene *PLVAP*, which is located downstream from tetherin in *P. alecto* (Fig. 4), is tetherin-like in that it encodes a protein with the same structural features as tetherin with the exception of a GSP (29). The human PLVAP protein does not possess restrictive activity against HIV-1 VLPs, but gains restrictive ability when modified to include a GPI-anchor (29). Sheep and cows possess a duplication of the tetherin gene (41, 53). In sheep, the duplicate tetherin, named tetherin B, similarly does not encode a GSP, and studies of sheep tetherin B function have revealed that while it is capable of limited restriction of VLPs, it is significantly less potent than sheep tetherin A (53). Its C-terminal amino acid sequence is entirely dissimilar from that of *P. alecto* tetherin isoform C. Interestingly, it has been demonstrated that substitution of the GSP with a transmembrane region in human tetherin produces a chimeric protein that retains the ability to inhibit viral particle release (64). However, the C-terminal region of *P. alecto* isoform C is not computationally predicted to include a transmembrane region, as determined using a transmembrane helix hidden Markov model, TMHMM 2.0 (65). One possible function of additional isoforms of tetherin is to expand the viral target range of bats by undefined mechanisms that are active in the absence of a GPI anchor.

Intriguingly, one of the non-transmembrane domain-associated sites under strong positive selection across all tetherins from bats is located in the middle of the extracellular domain. This is the site that distinguishes the *P. alecto* tetherin isoforms A and B, with the 7-aa exclusion at this location in isoform B. This suggests that the expression of *P. alecto* tetherin isoform B, and possibly also that of *M. macropus* tetherins B and E, which differ from tetherin A through a large 60-aa deletion in the same region, is to express a tetherin variant lacking sequence motif that appears to be a possible site of viral antagonism. This hypothesis could be supported by future experiments aimed at identifying viral antagonists of *P. alecto* tetherin isoform A that are ineffective against isoform B.

We found that the microbat *M. macropus* expresses five different tetherin genes, tetherins A, B, C, D, and E, of which three (tetherin A, C, and D), encode homologs of human l-tetherin, while *M. macropus* tetherin B and E possess a large 60-aa deletion within the extracellular coiled-coil domain (Fig. 4). Mapping of these genes against the genome assembly of the relatively distantly related M. lucifugus indicates that vesper bats of the genus *Myotis* may possess as many as seven tetherin genes (Fig. 6). The presence of multiple tetherin genes suggests that these bats have expanded and diversified their use of these antiviral restriction factors relative to other mammals, supporting the hypothesis of differential antiviral gene evolution in bats.

Tetherin was expressed widely and variably across the tissues of *P. alecto*, which is consistent with previous observations of human tetherin (36). We observed the highest levels of *P. alecto* tetherin expression in the thymus, possibly reflecting the role of the thymus in expressing a large proportion of the proteome due to its role in central tolerance. High expression levels were also observed in lung tissue, particularly in the lung of an individual female bat. This may reflect a frontline defensive role of tetherin in the lung against enveloped viruses which are respiratory pathogens.

Tetherin was upregulated in *P. alecto* spleen tissue treated with TLR agonists LPS and PIC. Interestingly, we observed that the expression of isoforms A, B, and C was highly variable among individual spleen samples. In several spleens, only isoform B was expressed, while in one spleen treated with PIC the vast majority of expression was attributed to isoform A. The remainder expressed variable levels of isoforms A and B, with the majority of expression trending toward isoform B. Surprisingly, isoform C was only expressed, at a low level, in a single spleen sample that had been treated with PIC. The biological importance of this observation is not presently known. Ongoing assessments of bat tetherin expression should determine whether this strong bias in alternative isoform expression is present in other tissues, such as the thymus and lung.

The expression of the *P. alecto* tetherin isoforms A, B, and C, and *M. macropus* tetherins A and B, reveals differences in their relative capacities to form homodimers under our assay conditions (Fig. 9A). This observation is notable because the ability of tetherin to form cysteine-linked dimers is required for the restriction of HIV-1 viral particles (59), which we chose as the VLPs against which bat tetherin proteins were functionally validated for inhibiting viral particle release. In contrast to restriction of HIV-1, tetherin dimerization is not required for the restriction of arenaviruses and filoviruses (66), suggesting that the need for dimerization is virus-dependent.

The *P. alecto* tetherin isoform A, which is homologous to human l-tetherin, was found to predominantly form dimers (Fig. 9A), which is consistent with human tetherin (Fig. 9B). In contrast, *P. alecto* tetherin isoforms B and C and *M. macropus* tetherin A were also observed in monomeric form under non-reducing conditions (Fig. 9A). *P. alecto* isoforms B and C contain a 7-aa exclusion in their ectodomain relative to isoform A, and isoform C possesses a distinct C-terminal region, which may account for this observation by potentially impacting dimer formation and/or stability. Additionally, we cannot currently rule out the possibility that the insertion of the HA tag influences tetherin dimerization. *M. macropus* tetherin B was observed as a dimer despite the absence of the conserved cysteine residues required for dimerization in the human tetherin homolog (Fig. 5A and Fig. 9A). The dimerization of *M. macropus* tetherin B may be due to the presence of additional cysteine residues at alignment positions C7, C10, and C118 (Fig. 5A). Bat tetherin proteins were observed to be less sensitive to reduction by dithiothreitol compared to human tetherin (Fig. 9C). One possible explanation for this is the predicted presence of a region of hydrophobicity at the N terminus (position 1 to 10 aa) of *P. alecto* and *M. macropus* that was not predicted for human tetherin (Fig. 10); however, other differences in the secondary structure of bat versus human tetherin may also account for this effect.

Previous reports on the necessity of the tetherin GPI anchor for inhibition of viral particle release (28, 43) indicate that the lack of a GPI anchor on *P. alecto* isoform C would likely result in an inability to inhibit viral egress from the host cell. However, sheep tetherin B, which does not possess a GPI-signal peptide (Fig. 2B), is capable of restricting the release of betaretroviruses by blocking glycoprotein incorporation into nascent virions (53, 67). Because of the unique sequence of the C terminus and lack of GSP in *P. alecto* tetherin isoform C, we extended our evaluation of its restrictive capacity to include filoviral VLPs derived from Ebola and Marburg virus VP40 matrix proteins. Surprisingly, *P. alecto* tetherin isoform C was capable of inhibiting the egress of Ebola VLPs to an extent similar to isoforms A and B, although it was less restrictive of Marburg VLPs compared to isoforms A and B (Fig. 13).

How isoform C could be capable of restricting the release of VLPs without a C-terminal GPI anchor is not presently known, although there are at least two possible explanations. The first is that the alternative C-terminal sequence of isoform C is involved in envelope or cellular membrane binding through an unknown mechanism. The second is that isoform C forms dimers in a manner distinct from that of isoforms A and B. One possibility is that it can form an antiparallel homodimer configuration in such a way that the N terminus of one monomer aligns with the C terminus of another, forming a dimer with a transmembrane domain at each end of the protein. Another possibility is the formation of standard parallel dimers of tetherin followed by the formation of an antiparallel tetramer, similarly resulting in a quaternary structure possessing a transmembrane domain at either end. This possibility is supported by structural analyses demonstrating that human tetherin can form tetramers composed of antiparallel pairs of parallel dimers (58, 68).

### Conclusions.

Tetherin variants of the fruit bat *P. alecto* and the vesper bat *M. macropus* are functionally capable of restricting the release of VLPs in a mammalian cell culture system. These bats have evolved to express unique forms of tetherin that do not conform to the standard protein structure observed in tetherin proteins of other mammals. A structurally unique tetherin of *P. alecto*, isoform C, was not observed to inhibit the egress of HIVΔVpu VLPs but was capable of restricting filoviral VLPs. These findings raise questions regarding the possible antiviral range, mechanism of action, and *in vivo* function of structurally diverse forms of tetherin. Bat tetherin genes are under strong positive selection, indicating an ongoing process of evolution involving tetherin targets and viral antagonists. This evolutionary pressure has resulted in the expansion and diversification of tetherin genes in vesper bats of the genus *Myotis*, and the emergence of unique splice variants of tetherin in the fruit bat *P. alecto*, supporting the hypothesis of differential and unique antiviral gene evolution in bats.

## MATERIALS AND METHODS

### Sequence read archive (SRA) BLAST analysis.

To predict the tetherin sequences of other bat species, the *P. alecto* tetherin isoform A coding domain nucleotide sequence (549 nt) was used as the search query for a separate BLASTn analysis of each of the publicly accessible sequence read archives (SRA) of bat transcriptomes (Table 1). This approach allowed the individual assembly of a tetherin homolog from each bat species for which a transcriptome sequence read archive was available. The BLASTn analyses were performed using the online SRA Nucleotide BLAST tool (https://blast.ncbi.nlm.nih.gov/Blast.cgi). The program selection was optimized for ‘somewhat similar sequences’ (BLASTn) and the selected algorithm parameters were as follows: max target sequences = 1,000; expect threshold = 1.0 × 10^−10^; word size = 7; max matches in a query range = 21; match/mismatch scores = 2,-3; gap costs of existence 5 and extension 2; and no filtering or masking was selected. Matching reads were downloaded and assembled using the Assemble Sequences tool in CLC Genomics Workbench 8.0 (CLC; Qiagen, Hilden, Germany, https://www.qiagenbioinformatics.com/). The consensus sequence of the assembly was then used as the search query for a second SRA BLAST analysis with the same parameters as the first search, with the following exceptions: program selection was optimized for ‘highly similar sequences’ (megablast); expect threshold = 1.0 × 10^−20^; word size = 28; match/mismatch scores = 1,-2; and linear gap costs of existence and extension. Matching reads were downloaded and assembled in the same manner, and the assembled consensus sequence was used in a third SRA BLAST using the same parameters as the second. This process was iteratively repeated for the assembled consensus sequence of each bat tetherin until it extended through the tetherin coding domain in both directions into the 5′ and 3′ untranslated regions, respectively demarcated by the locations of the start methionine and stop codon.

### Evolutionary selection test.

To determine if evolutionary selective pressures were being applied to bat tetherin, a selection test was performed. To detect the positively selected sites among bat tetherin sequences, a maximum likelihood (ML) phylogeny was generated with CODEML implemented in PAML4 software (69). The input tree was generated based on phylogeny generated by TimeTree (70). The coding sequence alignments were fit to the NSsites models allowing (M8; positive selection model) or disallowing (M8a; null model) positive selection. Models were compared using a chi-squared test (degrees of freedom = 2) on twice the difference of likelihood values to derive *P* values. The codon frequency model F3x4 was used. In cases where a significant difference (*P* < 0.01) between M8a and M8 was detected, a Bayes Empirical Bayes (BEB) analysis was used to identify codons with a ω (dN/dS) of >1, reporting values with a posterior probability of >0.99 as high significance or 0.95 to 0.99 as moderate significance.

### Identification of protein domains, structures, and motifs.

To confirm the predicted tetherin nucleotide sequences and identify functional domains within bat tetherin protein sequences, translated coding domains were compared against known tetherin amino acid sequences, including the human l-tetherin and the *P. alecto* tetherin isoform A (Fig. 2). For all tetherin amino acid sequences, secondary structures were determined using the Predict Secondary Structure tool in CLC. The presence of cytoplasmic, transmembrane, and extracellular domains was determined using a hidden Markov model in TMHMM 2.0 (65). GPI signal peptides (GSP) were determined using the online tool PredGPI (71) (http://gpcr2.biocomp.unibo.it/gpipe/index.htm). Multiple sequence alignments (MSA) of predicted and known tetherin amino acid and nucleotide sequences were performed using MUSCLE v3.8 (72) with the following parameters: maximum iterations = 16, find diagonals = no. The molecular weights of unglycosylated tetherin proteins with the GSP removed were calculated using the Expasy Compute pI/*M*_w_ online tool (73) (https://web.expasy.org/compute_pi/), with the addition of 1.5 kDa to account for the presence of the GPI anchor (74). Glycosylation sites were predicted using the GlycoMine online tool (50). Tetherin protein hydrophobicity was analyzed using the hydrophobicity plot tool in CLC with the following parameters: applied scale = Engleman; window size = 11.

### Transcriptome and contig analysis.

Approval for the use of bat tissue was granted by the Australian Centre for Disease Preparedness (ACDP) (formerly the Australian Animal Health Laboratory, AAHL) Animal Ethics committee (protocol no. AEC1281). The *P. alecto* transcriptome is accessible through the NCBI Sequence Read Archive (http://www.ncbi.nlm.nih.gov/Traces/sra/) (SRA: SRP008674).

To identify the homologs of tetherin in *P. alecto*, the human l-tetherin protein sequence (UniProt: Q10589-1) was used as a query in a tBLASTn analysis of the transcriptome of *P. alecto*. To identify assembled sequences (contigs) representing mRNA transcripts of interest, local tBLASTn analyses of the transcriptome of *P. alecto* were conducted with CLC using the following parameters: BLOSUM62 matrix, word size = 3, E values < 1 × 10^−12^, gap costs of existence = 11, extension = 1, with no filtering of regions of low complexity.

### cDNA analysis.

To amplify tetherin nucleotide sequences from bat cDNA, PCR assays were performed using cDNA generated from *P. alecto* and *M. ricketti* spleen tissue and *M. macropus* and *M. schreibersii* kidney cell lines, using various combinations of forward and reverse primers (Table 3). Primers were designed using the tetherin predictions identified in the contig and SRA analyses. Primers were designed to bind to the 5′ and 3′ untranslated regions of bat cDNA such that the full coding DNA sequence (CDS) could be amplified. Bat capture, tissue collection, and RNA extraction were conducted as previously reported (75), except that RNAlater (Ambion, Austin, TX)-preserved spleen tissue from four male adult bats was pooled before tissue homogenization followed by total RNA extraction with the Qiagen RNeasy Minikit with on-column digestion of genomic DNA using Dnase I. Total RNA was reverse transcribed into cDNA with the Qiagen Omniscript RT kit according to the manufacturer’s protocol, except that the reaction contained 100 ng/μL total RNA, 1 μM oligo-dT18 (Qiagen), and 10 μM random hexamers (Promega, Fitchburg, WI).

All PCR amplification assays were performed using the Roche FastStart High Fidelity PCR system (cat no. 04738292001) with an annealing temperature gradient of 54°C to 64°C in 2-°C increments. Each reaction was made up to a total of 20 μL containing 1 unit of polymerase, 2 ng of total cDNA, and 8 pmol of each primer. All other parameters for PCR amplification were set according to the manufacturer’s protocol.

Amplicons from PCRs were analyzed by agarose gel electrophoresis (76). Individual DNA bands were physically excised from the gel. DNA was purified from the gel fragments using the Wizard SV Gel and PCR Clean Up kit (Promega, Fitchburg, WI) according to the manufacturer’s protocol.

To further analyze the PCR-amplified DNA, each DNA fragment was blunt-end ligated into the pCR2.1-TOPO-TA or pCR-Blunt-II-TOPO plasmid vector (Invitrogen, Waltham, MA). Ligation was performed using the TOPO TA or Zero Blunt TOPO PCR Cloning kit (Invitrogen) according to the manufacturer’s instructions and plasmids were transformed into Top10 E. coli using a standard heat-shock method (76). Plasmids were purified from these cultures using a Wizard Plus SV Miniprep DNA Purification kit (Promega). All inserted sequences were confirmed by Sanger sequencing using M13 forward and reverse primers (M13F and M13R; Table 6).

**TABLE 6:** Sequencing primers used to confirm the sequence of tetherin clones and modified tetherin constructs inserted into plasmid vectors

Primer	Sequence (5′ > 3′)	Direction	Target region
M13F	GTAAAACGACGGCCAG	Forward	pCR-Blunt-II-TOPO vector
M13R	CAGGAAACAGCTATGAC	Reverse	pCR-Blunt-II-TOPO vector
T7F	TAATACGACTCACTATAGGG	Forward	pcDNA3.1 vector
BGHR	TAGAAGGCACAGTCGAGG	Reverse	pcDNA3.1 vector

### Genome mapping.

To identify tetherin genes within the genomes of *P. alecto* and *M. macropus*, local BLASTn analyses were performed using CLC. For these analyses, the *P. alecto* and *M. macropus* tetherin sequences were used as query sequences against the *P. alecto* (NCBI: PRJNA171993) and M. lucifugus genomes (NCBI: GCF_000147115), respectively. BLASTn was performed using the following parameters: word size = 11, E values < 1 × 10^−3^, gap costs of existence = 5, extension = 2, with no filtering of regions of low complexity. Genes were delineated as beginning and ending at the first and last nucleotides of the contig query sequence. Exons were delineated as consisting of the nucleotide regions within the gene mapping to the tetherin cDNA sequences, bordered by the canonical 5′-AG and 3′-GT dinucleotide motifs (77). The 5′ and 3′ untranslated regions were defined as the regions upstream of the start methionine and downstream of the stop codon of the CDS, respectively. Contig sequences were mapped against gene scaffolds through a local BLASTn analysis using CLC Genomics Workbench with default settings. The nucleotide sequence preceding the first exon of each gene was assessed for the presence of promoter motifs.

Neighboring genes were identified by BLAT (BLAST-like alignment tool) analysis (78) of the bordering nucleotide sequences upstream and downstream of tetherin genes using the Ensembl genome database BLAT tool (http://asia.ensembl.org/Multi/Tools/Blast?db=core).

### Generation of tagged tetherin constructs for expression in mammalian cells.

To enable detection of tetherin protein expression, the *P. alecto* tetherin isoforms A, B, and C and *M. macropus* tetherins A and B were genetically modified with the insertion of nucleotide sequences encoding the HA antibody-binding epitope. Tetherin sequences were modified through a two-step PCR process. PCRs were performed using the Roche FastStart High Fidelity PCR kit according to the manufacturer’s recommendations.

To express tetherin proteins in a mammalian cell culture system the tagged tetherin inserts were subcloned from the pCR2.1-TOPO (*P. alecto* tetherin isoforms A, B, and C) and pCR-Blunt-II-TOPO (*M. macropus* tetherin A and B) vectors into pcDNA3.1 mammalian expression vectors. The HA-tagged tetherin constructs contained terminal enzyme restriction sites. *P. alecto* tetherin constructs contained *Xho*I and *Xba*I sites at their 5′ and 3′ ends, respectively. *M. macropus* tetherin constructs contained an *Eco*RI site at each end. These sites, which are also present in the pcDNA3.1 vector, were used for digestion-ligation transfer of the HA-tagged tetherin sequences into pcDNA3.1. The ligation reaction products were transformed into Top10 E. coli and plasmid clones were purified from E. coli colonies using the method described in the ‘cDNA analysis’ section. All inserted sequences were verified by Sanger sequencing using the pcDNA3.1 vector sequencing primers T7F forward and BGHR reverse (Table 6).

### Expression, extraction, and detection of tetherin in a mammalian cell culture system.

To determine whether tetherin plasmids express tetherin protein in a mammalian cell culture system, adherent human embryonic kidney HEK293T cells (kindly provided by Richard Axel, Columbia University) were transfected with each tetherin construct with protein expression determined by Western blotting.

HEK293T cells were maintained in Dulbecco’s modified Eagle medium (DMEM-10, Thermo Fisher Scientific, Waltham, MA) enriched with heat-inactivated fetal calf serum (100 ml/L; Invitrogen), glutamine (292 μg/mL; Invitrogen), and the antibiotics penicillin (100 units/mL; Invitrogen) and streptomycin (100 μg/mL; Invitrogen) (DMEM-10). Cells were incubated at 37°C with 5% CO_2_.

The expression was performed in 6-well plates. Each well was seeded with 3 × 10^5^ cells/well in 2 mL DMEM-10. Cells were transfected when the monolayer had reached 50% to 60% confluence. The tetherin constructs analyzed are listed in Table 7, except for the human tetherin construct, pTethHA463, which has been previously described (60). Cells were transfected with each plasmid in duplicate wells at 2 μg/well using Lipofectamine 2000 (Thermo Fisher Scientific) according to the manufacturer’s protocol. Tetherin was extracted using a previously published GPI-anchored protein extraction protocol (49).

**TABLE 7:** Primers used for the generation of hemagglutinin-tagged *Pteropus alecto* and *Myotis macropus* tetherin expression constructs through a two-step PCR process

Tetherin	Construct	Two-step PCR usage	Primer		Direction	Encoded RE site	Encoded epitope
			Name	Sequence (5′ > 3′)			
Pteropus alecto							
Isoform A	pD-PaTAH1	Step 1, 5′ half	TethXF_JH_353	CTCTCTCGAGAGCTTCTTCTCCTGACTCC	Forward	*Xho* I	-^*a*^
			TethAB-HA1_JH_355	CGTATGGGTACCCGCTGGCATGCTCTTTCCTTAGCTG	Reverse	-	HA
		Step 1, 3′ half	TethAB-HA2_JH_356	CAGCGGGTACCCATACGATGTTCCAGATTACGCTGGCAGCTCTGGCGAGAAAAATGG	Forward	-	HA
			TethXR_JH_354	TTTTTCTAGAATGTTTCTCCACCCCTAAGGC	Reverse	*Xba* I	-
		Step 2	TethXF_JH_353	CTCTCTCGAGAGCTTCTTCTCCTGACTCC	Forward	*Xho* I	-
			TethXR_JH_354	TTTTTCTAGAATGTTTCTCCACCCCTAAGGC	Reverse	*Xba* I	-
Isoform B	pD-PaTBH1	Step 1, 5′ half	TethXF_JH_353	CTCTCTCGAGAGCTTCTTCTCCTGACTCC	Forward	*Xho* I	-
			TethAB-HA1_JH_355	CGTATGGGTACCCGCTGGCATGCTCTTTCCTTAGCTG	Reverse	-	HA
		Step 1, 3′ half	TethAB-HA2_JH_356	CAGCGGGTACCCATACGATGTTCCAGATTACGCTGGCAGCTCTGGCGAGAAAAATGG	Forward	-	HA
			TethXR_JH_354	TTTTTCTAGAATGTTTCTCCACCCCTAAGGC	Reverse	*Xba* I	-
		Step 2	TethXF_JH_353	CTCTCTCGAGAGCTTCTTCTCCTGACTCC	Forward	*Xho* I	-
			TethXR_JH_354	TTTTTCTAGAATGTTTCTCCACCCCTAAGGC	Reverse	*Xba* I	-
Isoform C	pD-PaTCH1	Step 1, 5′ half	TethXF_JH_353	CTCTCTCGAGAGCTTCTTCTCCTGACTCC	Forward	*Xho* I	-
			TethC-HA1_JH_359	TATGGGTACCCGCTCCTTAGCTGTTCCGGCTCCG	Reverse	-	HA
		Step 1, 3′ half	TethC-HA2_JH_360	AGCGGGTACCCATACGATGTTCCAGATTACGCTGGCAGCCCACAACCTGGACTGGTCC	Forward	-	HA
			TethXR_JH_354	TTTTTCTAGAATGTTTCTCCACCCCTAAGGC	Reverse	*Xba* I	-
		Step 2	TethXF_JH_353	CTCTCTCGAGAGCTTCTTCTCCTGACTCC	Forward	*Xho* I	-
			TethXR_JH_354	TTTTTCTAGAATGTTTCTCCACCCCTAAGGC	Reverse	*Xba* I	-
Myotis macropus							
Tetherin A	pD-MmTAH1	Step 1, 5′ half	MmacXF1_JH_510	CTGCAGAATTCGCCCTTATG	Forward	*Eco* RI	-
			MmacAH1_JH_512	GGGACGTCGTATGGGTATGGGTATGGCCGGCCGACCAAGGCCTCATTCTC	Reverse	-	HA
		Step 1, 3′ half	MmacAH2_JH_513	TACCCATACGACGTCCCAGACTACGCTGCTAGCTCTGCCAAGGGTCCCC	Forward	-	HA
			MmacXR1_JH_511	CAGTGTGCTGGAATTCGCC	Reverse	*Eco* RI	-
		Step 2	MmacXF1_JH_510	CTGCAGAATTCGCCCTTATG	Forward	*Eco* RI	-
			MmacXR1_JH_511	CAGTGTGCTGGAATTCGCC	Reverse	*Eco* RI	-
Tetherin B	pD-MmTBH1	Step 1, 5′ half	MmacXF2_JH_516	CTGCAGAATTCGCCCTTTCC	Forward	*Eco* RI	-
			MmacBH1_JH_518	CTGGGACGTCGTATGGGTATGGGTATGGCCGGCCGTTGGGGTCCTTGCCGAACAC	Reverse	-	HA
		Step 1, 3′ half	MmacBH2_JH_519	CCCATACGACGTCCCAGACTACGCTGCTAGCAATGGCAAGGGCTTCCCTAAC	Forward	-	HA
			MmacXR2_JH_517	CAGTGTGCTGGAATTCGCC	Reverse	*Eco* RI	-
		Step 2	MmacXF2_JH_516	CTGCAGAATTCGCCCTTTCC	Forward	*Eco* RI	-
			MmacXR2_JH_517	CAGTGTGCTGGAATTCGCC	Reverse	*Eco* RI	-

a The dashes indicate that either there is no encoded RE site, or no encoded epitope within the primer sequence referenced by that row.

Protein samples were analyzed by size-based separation through SDS-PAGE using either 12% polyacrylamide gels or Any kD gradient polyacrylamide gels (Bio-Rad, Hercules, CA) (76). Samples run under reducing and deglycosylating conditions were reduced by treatment of the samples with dithiothreitol (final concentration, 100 mM) and deglycosylated with PNGase F (New England Biolabs, Ipswich, MA), respectively, following the manufacturer’s instructions. Proteins were transferred to Amersham Protran nitrocellulose membranes (Sigma-Aldrich, St. Louis, MO) for Western blot analysis. Revert Total Protein stain (LI-COR Biosciences, Lincoln, NE) was applied to the membranes and visualized using the Odyssey Imaging System (LI-COR) according to the manufacturer’s protocol. For the Western blot analysis, the primary antibody solution contained a 1/1,000 dilution of a monoclonal rabbit anti-HA antibody (C29F4, Cell Signaling Technology, Danvers, MA) in Tris-buffered saline (TBS [pH 7.6]) containing 0.1% Tween 20, and the secondary antibody solution contained a 1/10,000 dilution of a polyclonal goat anti-rabbit IRD800 fluorophore-conjugate secondary antibody (LI-COR). The primary antibody solution was incubated overnight at 4°C, and the secondary antibody solution was incubated at room temperature for 1 h. To visualize fluorescent antibody-bound tetherin proteins, membranes were scanned using the Odyssey Imaging System (LI-COR) at wavelengths of 600 and 800 nm, using the default software settings.

### Fluorescence microscopy.

To visualize tetherin localization within cells, 500 ng of plasmids encoding HA-tagged human l-tetherin and *P. alecto* tetherin isoforms A, B, and C was transfected into HEK293T cells seeded on glass coverslips as described above. At 48 h post-transfection, cells were fixed with 4% paraformaldehyde (Sigma) in phosphate-buffered saline (PBS) for 10 min at room temperature, and then permeabilized in 0.2% Triton X-100 in PBS for 5 min at room temperature. Tetherin localization in cells was detected by staining cells with anti-HA-tag rabbit monoclonal IgG (Thermo Fisher) diluted in 0.2% Triton X-100, 3% bovine serum albumin (BSA) in PBS for 1 h at room temperature. Cells were subsequently stained with anti-rabbit Alexa Fluor 488 secondary IgG (Thermo Fisher) diluted in 0.2% Triton X-100, 3% BSA in PBS for 20 min at room temperature. Nuclei were blue-stained with Hoechst 33342 (Thermo Fisher) according to the manufacturer’s instructions. Coverslips were mounted onto glass slides with ProLong Gold Antifade Mountant (Thermo Fisher), then imaged on a Nikon AR1 confocal microscope and analyzed with ImageJ (NIH) software.

### qPCR analysis of tetherin expression across multiple bat tissues.

To determine tetherin expression across various *P. alecto* tissues, tetherin mRNA levels were measured by qPCR analysis (Table 8). The primers and probes were designed using the program Primer Express (Perkin-Elmer, Applied Biosystems, Waltham, MA). The tetherin primers amplify all three known isoforms of *P. alecto* tetherin. *P. alecto* bats were trapped in Queensland, Australia, and transported alive by air to the ACDP in Victoria, where they were euthanized for dissection using methods approved by the ACDP animal ethics committee (AEC1389). Tissues were stored at −80°C in RNAlater (Ambion). Total RNA was extracted from frozen *P. alecto* tissues using a Precellys 24 tissue homogenizer (Bertin Technologies, France) and a RNeasy minikit (Qiagen) with on-column DNase-I treatment (Qiagen) to remove traces of genomic DNA.

**TABLE 8:** qPCR primers and probes used to analyze tetherin expression in bat tissues^*a*^

Primer or probe	Target	Sequence (5′ > 3′)^*b*^	Direction
Primers			
Teth_F	Tetherin	TGACTGTGGCCGTGATCGT	Forward
Teth_R		CCATTTTTGCAGGCCTCACT	Reverse
18S_rRNA_F	18S rRNA	CGGCTACCACATCCAAGGAA	Forward
18S_rRNA_R		GCTGGAATTACCGCGGCT	Reverse
Probes			
Teth_Probe	Tetherin	*FAM*_TCGCCGTCGAGAACA_*MGB*	NA
18S_Probe	18S rRNA	*VIC*_TGCTGGCACCAGACTTGCCCTC_*TAMRA*	NA

a qPCR, quantitative PCR; NA, not applicable.

b Probe 5′ reporters and 3′ quenchers are indicated with italics.

Total RNA was subjected to real-time PCR using SuperScript III one-step RT-PCR System (Invitrogen) with Platinum *Taq* DNA polymerase. Reactions were performed on 100 ng of template RNA with 200 nM each primer and 150 mM the TaqMan probe in an Applied Biosystems 7500 Fast Real-Time qPCR instrument. Cycling parameters consisted of 50°C for 5 min for the reverse transcription of RNA to cDNA followed by 95°C for 2 min. The cDNA was amplified by PCR for 40 cycles, each consisting of 95°C for 3 s and 60°C, 30 s. Using the Livak/ΔΔ*C_T_* method (79), tetherin *C_T_* values were normalized against the expression/*C_T_* values of the 18S rRNA housekeeping gene and reported as fold-difference, calibrated against the *C_T_* values for wing tissue.

### Transcriptome analysis of isoform expression under immune-stimulating treatments.

To compare the expression of alternative isoforms of *P. alecto* tetherin and the impact of treatment with immune-stimulating compounds, bats were treated with PBS, LPS (InvivoGen, San Diego, CA), or PIC (InvivoGen) as described previously (47). Briefly, 5 h post-intraperitoneal injection, bats were anesthetized and culled and organs were processed for RNA, DNA, protein, and cell suspensions as described previously (80). RNA libraries were prepared using RiboZero Plus rRNA-depletion kits (Illumina, San Diego, CA) and cDNA was generated using a mix of oligo-dT/random hexamer primers, prior to sequencing in 2×150PE on the Illumina HiSeq platform.

Sequencing read libraries were quality controlled by analysis using FastQC (81). Illumina sequence adapters were removed and reads were trimmed or discarded on the basis of quality using the Trim Sequences tool in CLC. Overlapping paired reads were merged using the Merge Overlapping Pairs tool in CLC. Using the RNA-Seq Analysis tool in CLC, sequence reads were mapped against the *P. alecto* gene scaffold containing the tetherin gene (GenBank accession no. KB030270.1), which was manually annotated with the tetherin gene and mRNA sequences for isoforms A, B, and C. The following parameters were used for the RNA-Seq analysis: mismatch = 3, insertion = 3, deletion = 3, length fraction = 0.6, and similarity fraction = 0.95; default parameters were used otherwise. Isoform expression levels were normalized for post-trimming read library size and reported as counts-per-million reads (CPM). Non-parametric one-tailed Mann-Whitney U tests were performed to calculate statistical significance between treatments. The *P. alecto* tetherin gene scaffold, annotation files, and read maps are provided in supplemental Data Set S2.

### Functional validation of tetherin activity.

To determine the inhibitory effect of bat tetherin on the release of virus-like particles, HEK293T cells were co-transfected with each tagged bat or human tetherin construct and either a HIVΔVpu plasmid construct, pCRV1-NlgagpolΔVpu, which expresses HIV NL4.3 Gag-polΔVpu protein (NCBI: P12497.4); EBOV VP40 plasmid, pGFPEVP40, expressing the Ebola Zaire virus VP40 Matrix protein (NCBI: NP_066245); or a MARV VP40 plasmid, pGFPEMVP40, expressing the Marburg virus VP40 Matrix protein (NCBI: YP_001531155). EBOV and MARV VP40 proteins were encoded and expressed as VP40-eGFP fusion proteins. VLP plasmid and human tetherin constructs were kindly provided by Paul Bieniasz (Aaron Diamond AIDS Research Center, Rockefeller University, New York, NY) (82).

Transfections were performed as described above, with the following modifications: 12-well plates were seeded with 1.5 × 10^5^ cells/well in a total volume of 1 mL DMEM-10, DNA-lipofectamine mixtures for each well contained 1 μL lipofectamine and a total of 400 ng DNA, comprised of 200 ng of VLP plasmid DNA and 0 to 200 ng of tetherin-encoding plasmid DNA, to a total mass of 200 ng with insert-free pcDNA3.1 plasmid DNA. Each sample was prepared in duplicate wells. Cells were incubated at 37°C with 5% CO_2_ for a total of 48 h. After 24 h, 1 mL DMEM-10 was added to each well. Following incubation, cell lysates and supernatants were collected for further analysis.

Cell culture supernatants were collected and clarified. Clarified supernatants were layered over 2 mL of a 25% (wt/vol) sucrose solution in 13.2 mL Thinwall Polypropylene SW41 Ti ultracentrifuge tubes (Beckman-Coulter, Inc., Brea, CA) made up to a total volume of 10 mL with PBS without calcium chloride or magnesium chloride (PBS[–]). Samples were then centrifuged at 130,000 × *g* for 2 h at 4°C in an Optima L-100 XP ultracentrifuge (Beckman-Coulter). VLP pellets were lysed in 60 μL of NP-40 lysis buffer containing 1% NP-40 (Sigma-Aldrich) and 10 μg/mL each of aprotinin, leupeptin, and pepstatin A protease inhibitors (Sigma-Aldrich).

The cells from each well were resuspended in 500 μL of PBS[–], transferred to 1.5 mL tubes, and centrifuged at 200 × *g* for 5 min. Cell pellets were lysed in 100 μL of NP-40 lysis buffer. Following cell lysis, samples were clarified by centrifugation at 20,000 × *g* for 5 min at 4°C, and supernatants were collected for SDS-PAGE.

Cell and viral lysates were analyzed by SDS-PAGE and Western blot analysis as described above, with the following exceptions. For HIV VLPs, the primary antibody binding was directed against the Gag protein using a solution containing a 1/4,000 dilution of a mouse anti-p24 antibody (NIH AIDS Reagent Repository, Germantown, MD) and 0.1% Tween 20 in PBS[–]. For Ebola and Marburg VLPs, the primary antibody binding was directed against eGFP using a solution containing a 1/4,000 dilution of a mouse anti-GFP 4B10 antibody (Cell Signaling Technology, Danvers, MA). For all VLPs, the secondary antibody was a 1/10,000 diluted polyclonal goat anti-mouse Alexa-Fluor 680 fluorophore-conjugate fluorescent secondary antibody (Thermo Fisher).

VLPs released into the supernatant for each sample were quantified using a densitometric analysis of the signal strength of the fluorescent antibodies bound to VLP proteins. Densitometry was performed using the Image Studio Lite v5.2 software suite (LI-COR). Variation in viral lysate signal strength across samples was normalized using the signal strength of VLP proteins in the cell lysates. A nonparametric Wilcoxon rank-sum test was performed to calculate the statistical significance of the restriction of Ebola and Marburg VLPs in the 200 ng tetherin isoform C treatment groups.

### Data availability.

The data underlying this article are available in this article and its online supplemental material. Novel tetherin sequences have been deposited in GenBank (https://www.ncbi.nlm.nih.gov/genbank/) under the accession numbers listed in Table 1.
